# Large-Scale Mutagenesis in *p19^ARF^*- and *p53*-Deficient Mice Identifies Cancer Genes and Their Collaborative Networks

**DOI:** 10.1016/j.cell.2008.03.021

**Published:** 2008-05-16

**Authors:** Anthony G. Uren, Jaap Kool, Konstantin Matentzoglu, Jeroen de Ridder, Jenny Mattison, Miranda van Uitert, Wendy Lagcher, Daoud Sie, Ellen Tanger, Tony Cox, Marcel Reinders, Tim J. Hubbard, Jane Rogers, Jos Jonkers, Lodewyk Wessels, David J. Adams, Maarten van Lohuizen, Anton Berns

**Affiliations:** 1 Division of Molecular Genetics and Cancer Genomics Centre, Netherlands Cancer Institute, Plesmanlaan 121, 1066CX, Amsterdam, The Netherlands; 2 Division of Molecular Biology, Netherlands Cancer Institute, Plesmanlaan 121, 1066CX, Amsterdam, The Netherlands; 3 Faculty of Electrical Engineering, Mathematics, and Computer Science, Delft University of Technology, 2628CD, Delft, The Netherlands; 4 Wellcome Trust Sanger Institute, Wellcome Trust Genome Campus, Hinxton, CB101SA, UK; 5 Central Microarray Facility, Netherlands Cancer Institute, Plesmanlaan 121, 1066CX, Amsterdam, The Netherlands

**Keywords:** SYSBIO, SIGNALING, HUMDISEASE

## Abstract

*p53* and *p19^ARF^* are tumor suppressors frequently mutated in human tumors. In a high-throughput screen in mice for mutations collaborating with either *p53* or *p19^ARF^* deficiency, we identified 10,806 retroviral insertion sites, implicating over 300 loci in tumorigenesis. This dataset reveals 20 genes that are specifically mutated in either *p19^ARF^*-deficient, *p53*-deficient or wild-type mice (including *Flt3*, *mmu-mir-106a-363*, *Smg6*, and *Ccnd3*), as well as networks of significant collaborative and mutually exclusive interactions between cancer genes. Furthermore, we found candidate tumor suppressor genes, as well as distinct clusters of insertions within genes like *Flt3* and *Notch1* that induce mutants with different spectra of genetic interactions. Cross species comparative analysis with aCGH data of human cancer cell lines revealed known and candidate oncogenes (*Mmp13, Slamf6,* and *Rreb1*) and tumor suppressors (*Wwox* and *Arfrp2*). This dataset should prove to be a rich resource for the study of genetic interactions that underlie tumorigenesis.

## Introduction

Retroviral insertional mutagenesis in mice has proven to be an efficient tool for identification of novel cancer genes, providing a valuable complement to the study of human tumors. Slow transforming retroviruses, such as Moloney Murine Leukemia Virus (MuLV), can mutate cellular genes by integration of their proviruses into the host genome. Cells that have acquired a proliferative advantage through proviral mutation of cellular genes, may acquire additional mutations by viral reinfection and eventually develop into tumors (reviewed in Uren et al., 2005). The position of insertions can be determined by amplifying DNA flanking the provirus using linker-mediated PCR and mapping the resulting sequences onto the genome. Many regions that are tagged in multiple independent tumors (termed Common Insertion Sites or Common Integration Sites or CISs) have been identified in previous studies (compiled in the RTCGD, http://rtcgd.abcc.ncifcrf.gov/, (Akagi et al., 2004)). A high proportion of these loci are orthologous with the loci of known human cancer genes, examples being *Fli-1*, *Evi1*, and *Pim1* (Ben David et al., 1988; Morishita et al., 1988; Cuypers et al., 1984). However, the oncogenic capacity of many of the candidate cancer genes identified by these screens still needs further experimental validation.

Components of the p19^ARF^-MDM2-p53 signaling pathway are mutated in a large fraction of human cancers (Sharpless, 2005; Levine et al., 2006). p19^ARF^ acts upstream of p53 and can enhance its transcriptional activity by antagonizing MDM2-mediated degradation of p53 (Sherr, 2006). Comutation of *p19^ARF^* and *p53* rarely occurs (Eischen et al., 1999; Schmitt et al., 1999), and deletion of *p19^ARF^* in heterozygous *p53* knockout mice reduces the selective pressure for loss of the *p53* wild-type allele for tumor formation (Moore et al., 2003). In addition, p19^ARF^ was shown to be pivotal for suppression of chemically- or radiation-induced tumors by p53 in mice (Efeyan et al., 2006; Christophorou et al., 2006). These data suggest the primary function of p19^ARF^ is to activate p53. However, several studies indicate that p19^ARF^ may also suppress tumorigenesis independently of p53. Mice lacking both *p19^ARF^* and *p53* showed tumors in a wider range of tissue types and more frequently developed multiple primary tumors than mice lacking either of the two genes (Weber et al., 2000). Furthermore, loss of *p19^ARF^* increased the number and size of chemically-induced papillomas both in wild-type and *p53^−/−^* mice (Kelly-Spratt et al., 2004).

We performed insertional mutagenesis screens in *p53^−/−^*, *p19^ARF−/−^* and wild-type mice to identify genes that collaborate with loss of either *p53* or *p19^ARF^* in tumorigenesis and in doing so gained new insight in the functional differences between these tumor suppressors. We also used these data to construct networks of collaborative and mutually exclusive interactions between CIS loci. To date, the primary limitation of identifying genotype-CIS and CIS-CIS collaborations has been not only the number of tumors but also the number of inserts per tumor. If only a fraction of the inserts of each tumor are detected, the power of any statistical test for genotype specificity or for collaboration between loci will be greatly reduced. With this in mind, we optimized our insertion site cloning and analyzed more than 500 tumors yielding over 10,000 independent insertion sites. By comparison, many of the screens published to date identify hundreds of insertions (Li et al., 1999; Hwang et al., 2002; Lund et al., 2002; Mikkers et al., 2002; Suzuki et al., 2002; Johansson et al., 2004; Theodorou et al., 2007; Stewart et al., 2007; Slape et al., 2007), the largest containing 1538 insertions from 245 tumors (Suzuki et al., 2006). As a result, a large number of novel CISs were identified near known and candidate oncogenes and tumor suppressor genes. These data create a resource (http://mutapedia.nki.nl) that will help cancer researchers to identify new cancer genes and further investigate the interactions of established oncogenic lesions.

## Results

### MuLV Accelerates Lymphomagenesis in *p19^ARF−/−^* and *p53^−/−^* Mice

**Figure 1:**
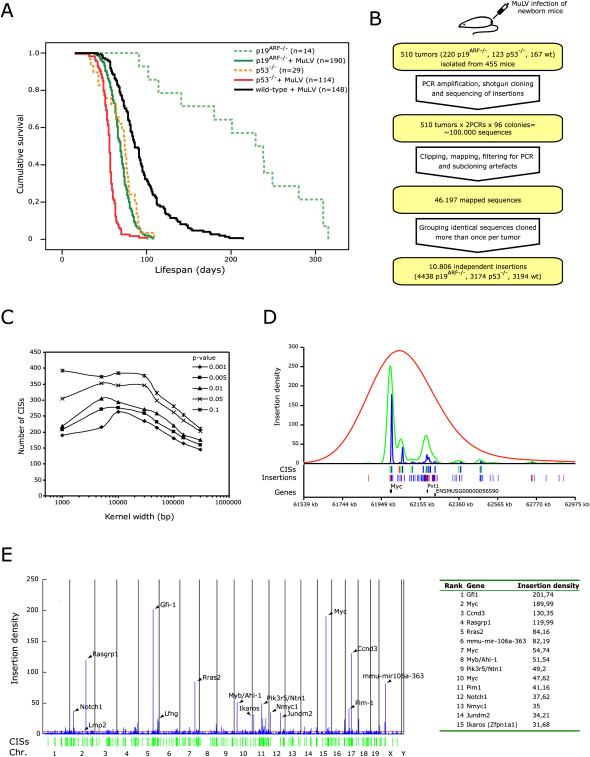
Accelerated Tumor Formation in MuLV-Infected Mice and Identification of Common Insertion Sites Using the GKC Framework (A) Cumulative survival of *p19^ARF−/−^*, *p53^−/−^*, and wild-type mice infected with MuLV and noninfected *p19^ARF−/−^* and *p53^−/−^* mice is plotted against lifespan in days (*p19^ARF−/−^* versus wild-type, p value < 0.0001; *p53^−/−^* versus wild-type, p value < 0.0001, log-rank test). (B) Flow chart for retrieving retroviral insertions. (C) Number of CISs varies with kernel size and significance. (D) Identification of CISs near *Myc* with different kernel sizes. Red line represents insertion density for 300 kb kernel, green line for 30 kb, and the blue line for 5 kb. Blue and red denote sense and antisense insertions, respectively. (E) Insertions from *p53^−/−^*, *p19^ARF−/−^*, and wild-type tumors were analyzed together with a 30 kb kernel to determine insertion density over the genome (left panel). The cut-off (p value < 0.05) for significant insertion density is indicated (red line). CISs (p value < 0.05) are indicated by green vertical bars. A list of the insertion density of the 15 most significant CISs is included (right panel).

We conducted retroviral insertional mutagenesis screens in *p19^ARF−/−^*, *p53^−/−^* and wild-type mice on a pure FVB genetic background. Mice were infected with MuLV at postnatal day 1 and monitored for tumor growth. Both *p53^−/−^* and *p19^ARF−/−^* mice are predisposed for development of lymphoma (Donehower et al., 1992; Kamijo et al., 1999), and MuLV infection further accelerated lymphomagenesis in these mice as well as in wild-type mice (p value < 0.0001 for MuLV-infected *p19^ARF−/−^* versus noninfected *p19^ARF−/−^*, p value < 0.0001 for MuLV-infected *p53^−/−^* versus noninfected *p53^−/−^*, log-rank test) (Figure 1A). Mice developed tumors almost exclusively in spleen, thymus and lymph nodes. The majority of tumors (n = 349) were analyzed by flow cytometry using T cell and B cell-specific markers (CD3 and B220, respectively, Figure S1A). No large differences were found in the incidence of tumors in these organs between the different genotypes, and the proportion of analyzed tumors from the different organs is roughly the same for each genotype (Figure S1B).

### High-Throughput Cloning and Sequencing of MuLV Insertion Sites

To identify genes mutated by proviral insertions, flanking sequences of the retroviral insertions were cloned by linker-mediated PCR (Devon et al., 1995; Mikkers et al., 2002) using tumor DNA digested with either *Sau3aI* or *Tsp509I*. For the majority of samples two PCRs were performed (one for each enzyme) and shotgun subcloned. 96 colonies per PCR were picked and sequenced. In total, 510 tumors (220 *p19^ARF−/−^*, 123 *p53^−/−^*, and 167 wild-type), isolated from 455 mice were analyzed, yielding approximately 100,000 sequence reads. After filtering, 46,197 could be mapped and oriented onto the genome. Sequences cloned more than once from the same mouse likely represent the same insertion. Therefore, these were built into contigs to avoid overcounting the insertions of disseminated tumors. In total, 10,806 independent insertions were retrieved (3194, 4438, and 3174 from wild-type, *p19^ARF−/−^* and *p53^−/−^* tumors, respectively) (for overview see Figure 1B), yielding an average of 23.7 insertions per mouse.

### Identification of CISs Using a Kernel Convolution-Based Statistical Framework

To identify common insertion sites (CISs), i.e., regions in the genome that are significantly more frequently mutated by insertions than would be expected by chance, we used a statistical framework based on Gaussian kernel convolution (GKC), which estimates a smoothed density distribution of inserts over the entire genome (Figure S2A) (de Ridder et al., 2006). Depending on kernel size and p value, the total number of statistically significant CISs varies (Figure 1C, Table S1). Increasing kernel size may result in merging independent CISs that may influence the same gene (as illustrated for the *Myc* locus in Figure 1D). Smaller kernels sizes may, conversely, reveal separate CISs that affect the same gene, which may be preferentially mutated in specific genetic backgrounds or result in expression of different mutant proteins (see below). Unless stated otherwise a kernel size of 30 kb was used in this paper.

### Large-Scale Identification of CISs Near Known and Unknown Cancer Genes

Applying the GKC framework to the 10,806 insertions from the *p53^−/−^*, *p19^ARF−/−^* and wild-type tumors identified 346 CISs (p value < 0.05) (Figure 1E). By comparison, similar analysis of the MuLV insertion data present in the RTCGD database identified 160 CISs from 5435 insertions, 54 of these being shared between these datasets (Figure S2B). Combined analysis of both datasets yielded 473 CISs. To select genes that are the most likely candidate cancer genes, we used the mechanisms of retroviral mutation described by Jonkers and Berns, 1996 (Table S2). Established proto-oncogenes like *Myc*, *Nmyc1*, *Ccnd3*, *Pim1* and *Notch1*, rank among the most significant CISs of this screen (Figure 1E, Table S2). In addition, established cancer genes like *Lmo2*, *RhoH*, *Trim33*, *Mll*, and *Hspca* (Futreal et al., 2004) are candidate target genes of less frequently mutated CISs, indicating that lower ranked CISs (carrying 4–5 insertions) may represent bona fide cancer genes. Importantly, we found a large number of highly significant CISs near genes that have not previously been linked to tumorigenesis, including *Pik3r5* and *Pik3cd*, both regulatory subunits of PI3K that upon retroviral activation may enhance PI3K signaling (Brock et al., 2003), miRNA genes like e. g. *mmu-mir-24-2/27a/23a* and *mmu-mir-142*, *Lunatic Fringe* (*Lfng*), a modulator of Notch receptor activity (Haines and Irvine, 2003) and *Smg6* (*Est1a*) which is involved in nonsense-mediated RNA decay (Fukuhara et al., 2005).

### CISs Enriched for Established Cancer Genes

To determine what proportion of our CISs corresponds to verified human cancer genes, we compared our CISs with lists of known cancer genes. The Cancer Gene Census is an actively curated list of genes the mutation of which contributes to tumor formation (Futreal et al., 2004). Another list of mutated genes has been derived from sequencing the coding regions of a panel of breast and colon tumors (Sjoblom et al., 2006). From these, we composed a list of 516 unambiguous murine orthologs of human cancer genes. Depending on window size around the CIS midpoint (+/− 50 kb to 300 kb), we found between 30–79 CISs in the vicinity of murine orthologs of human cancer genes, a highly significant enrichment compared to an equal number of randomly selected genes (p value ≈0 for all window sizes). This indicates that CISs are found in the vicinity of cancer genes more frequently than expected by chance. We then used this overlap as a measure of the saturation of our screen i.e., to see how many of these cancer genes might have been identified using fewer tumors. Subsets of the total dataset were selected by stepwise addition of randomly selected groups of 50 mice. CISs were then identified for these random subsets, and we determined the number of known cancer gene orthologs within 200 kb either side of each CIS midpoint. The number of cancer genes identified does not reach its maximum until all the tumors are included (Figure S3), indicating that significant numbers of known (and by extension unknown) cancer genes might still be found by performing more extensive studies with larger amounts of tumors.

### Insertions Mutate Pathways Involved in Human Cancer

To identify signaling pathways activated by retroviral insertions, we took the 346 candidate target genes and used Ingenuity Pathway Analysis software to investigate whether these genes play a role in canonical signaling pathways. Our 346 candidate genes were most significantly enriched for genes implicated in T cell and B cell receptor signaling, GM-CSF signaling and IL-2 signaling (Table S3), which may have been expected since all tumors were of lymphoid origin. In addition, we find significant enrichment for mutation of genes involved in ERK/MAPK, PI3K/AKT and G1/S checkpoint regulation. Genes in these pathways are also commonly mutated in human cancers, indicating that the signaling pathways mutated in our screen overlap with signaling pathways deregulated in human cancer (Table S3, Cancer Gene Census genes). Selection of the 346 candidate genes may be biased by manual curation. To rule out possible biases, we also compiled a list of the nearest gene to the midpoint of each of the 346 CISs. This yielded similar results, demonstrating that these results are not due to manual curation of CIS candidate target genes.

### Identification of Genes Collaborating with *p19^ARF^* or *p53* Deficiency

**Figure 2:**
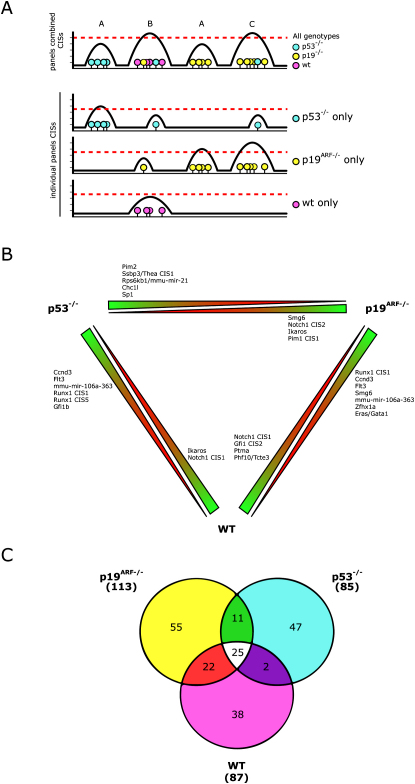
Genotype-Specific Distribution of Insertions (A) Analysis of all panels combined will detect CISs that occur relatively frequently (peaks B and C) but may not identify CISs in regions that have a relatively small number of insertions in tumors from the same genotype (peaks A). Conversely, CISs constituted of a number of insertions from different panels, may not reach significance when panels are analyzed separately (peak B). (B) Schematic overview of genotype-specific CISs found in *p19^ARF−/−^*, *p53^−/−^*, and wild-type mice. CISs that have a significant bias (p value < 0.05) toward a particular genotype are depicted. CISs more frequently found in one genotype versus another (e.g., Pim2 in *p53^−/−^* versus *p19^ARF−/−^*) are depicted at the green end of the wedge pointing toward the other genotype (*p19^ARF−/−^*). (C) Venn-diagram representing the overlap in numbers of “individual panel” CISs found between genotypes. The number of CISs per genotype is indicated outside the diagram.

To identify genes that specifically collaborate with deficiency for either *p19^ARF^* or *p53*, the dataset was analyzed in two ways: (a) CISs were determined using all the insertions from the three panels together and CIS-genotype interactions were identified by comparing the number of insertions in a CIS in one genotype versus another, or (b) CISs were determined per panel, and candidate genes of the CISs were compared between panels (see Figure 2A).

Using the first approach (a), with all panels combined, we found 21 CISs (20 genes) with a significant bias (p < 0.05) toward one of the genotypes (Figure 2B, Table 1). For example, *Runx1*, *Ccnd3*, the miRNA cluster *mmu-mir-106a-363* (encoding mmu-mir-106a/20b/19b-2/92-2 and 363), *Flt3* and *Smg6* are preferentially mutated in *p19^ARF−/−^* tumors compared to wild-type tumors. *Runx1*, *mmu-mir-106a-363*, *Ccnd3*, and *Flt3*, but not *Smg6*, are also specific for *p53^−/−^* compared to wild-type, indicating that mutation of these genes is selected for in cells that lack the p19^ARF^-p53 tumor suppression pathway. In contrast, mutation of *Smg6* is highly specific for *p19^ARF−/−^* (*p19^ARF−/−^* versus wild-type p value = 0.0047, *p19^ARF−/−^* versus *p53^−/−^* p value = 0.0003) and may therefore only contribute to tumorigenesis in a *p19^ARF^*-deficient background. P53 can inhibit tumor development in the absence of p19^ARF^. We find that Notch1, which is able to suppress p53 activity through a p19^ARF^-independent, MDM2-dependent pathway (Beverly et al., 2005), is more frequently mutated in wild-type and *p19^ARF−/−^* compared to *p53^−/−^* (Figure 2B), suggesting that activation of Notch1 in *p19^ARF−/−^* and wild-type tumors might be instrumental for suppression of p53 activity. Similar numbers of genes are genotype-specific for *p53^−/−^* compared to *p19^ARF−/−^* and *p53^−/−^* compared to wild-type. However, genes preferentially mutated in *p53^−/−^* versus wild-type collaborate more strongly with *p53* deficiency (yield lower p values) than genes preferentially mutated in *p53^−/−^* versus *p19^ARF−/−^*.

In our second approach (b), we determined CISs per panel, thus analyzing insertions that were retrieved from tumors isolated from mice with the same germ-line genotype. We find 113 CISs in *p19^ARF−/−^* tumors, 85 in *p53^−/−^* and 87 in wild-type tumors. The Venn diagram (Figure 2C) shows that 25 CISs near frequently mutated genes like *Myc*, *Gfi1*, *Rasgrp1* and *Rras2* are found in all panels (Figure 2C, Table S4). However, all panels also have a relatively large number of unique CISs, including 17 new CISs that were not found when insertions from the different panels were analyzed together. Fourteen of these newly identified loci are exclusively mutated in one of the three genotypes (Table S4). To determine the activated canonical signaling pathways, we analyzed candidate target genes of CISs unique for a single genotype (e.g., *p19^ARF−/−^* but not wild-type and *p53^−/−^*) with Ingenuity Pathway Analysis. We also analyzed all CISs that are found in one or two genotypes, but not the third (e.g., *p19^ARF−/−^* and/or *p53^−/−^* but not wild-type). We found that genes implicated in p53 signaling such as *Akt1*, *Bcl2l1*, *Gadd45b* and *Ccnd1* are mutated in wild-type or *p19^ARF−/−^* tumors but not in *p53^−/−^* tumors (Tables S4 and S5). Activation of Akt1 can induce MDM2-mediated degradation of p53 and may thereby suppress p53-dependent effects on tumor formation (Mayo and Donner, 2001). Bcl2l1 can inhibit p53-induced apoptosis (Eischen et al., 2001), whereas Gadd45 has been demonstrated to have anti-apoptotic activity in hematopoietic cells in response to genotoxic stress (Gupta et al., 2005). Thus, activation of these genes in wild-type and *p19^ARF−/−^* tumors may abrogate p53-mediated apoptosis and contribute to tumor formation.

### Creating an Interaction Map for All CISs

Previous analyses of insertions in the RTCGD have noted that some CISs appear to cooperate in oncogenesis (Dave et al., 2004; de Ridder et al., 2007). We looked for CIS-CIS interactions by determining whether particular CISs are found mutated together in the same tumor at higher or lower rates than expected by chance. We performed analysis of all pairs of CISs, using contingency tables that assume one CIS is the predisposing event, whereas inserts in the second CIS are assumed to be subsequent events. The reciprocal assumption was also tested.

**Figure 3:**
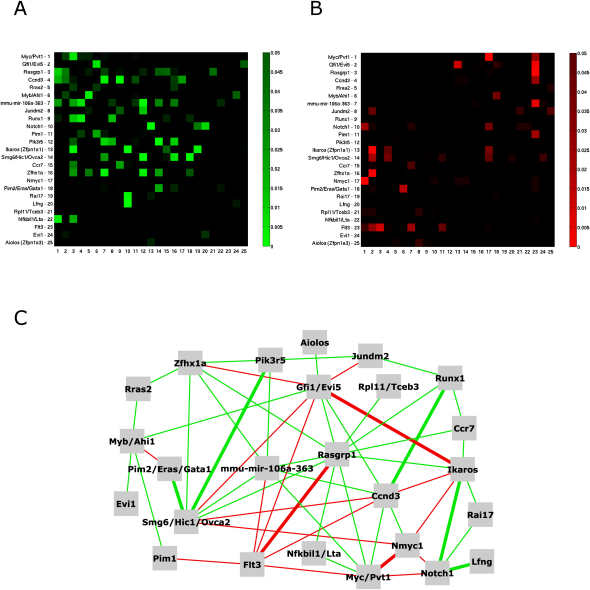
Mapping Interaction Networks between Common Insertion Sites (A) Co-occurrence between the top 25 300 kb CISs. CIS names and CIS rank are indicated on vertical axis, numbers on horizontal axis are CIS rank. The horizontal axis represents CISs that are assumed to be the predisposing, more clonal event and the vertical axis represents CISs that are presumed to be subsequent, subclonal events. (B) Mutual exclusivity between the top 25 300 kb CISs. Set up of the figure as described in (A). (C) CIS interaction network representing the co-occurrence or mutual exclusivity of the 20 most significant CISs. Co-occurring CISs are connected by green lines (thin line, 0.001 < p value < 0.05; heavy line, p value < 0.001), mutual exclusive CISs are connected with red lines (thin line, 0.001 < p value < 0.05; heavy line, p value < 0.001).

The set of interactions between all 300 kb CISs is depicted in Figure S4, with Table S6 ranking the most significant interactions by p value. Interactions between the top 25 300 kb CISs are depicted as a heat map in Figures 3A and 3B and as a network in Figure 3C. The symmetry observed over the diagonal of these plots indicates that the contingency table tests yield similar results regardless of which insert is assumed to have occurred earlier. Nonetheless, some CIS pairs do break from this trend; for instance, insertions mutating *Notch1* in the presence of an existing *Rai17* insertion (p value = 0.001) appear to be more strongly selected for than insertions mutating *Rai17* in the presence of an existing *Notch1* insertion (0.0356).

Several of these interactions have some precedent within the literature. For example, *Ikaros (Zfpn1a1)* and *Notch1* insertions frequently co-occur within the same tumor. Ikaros, a transcriptional regulator of hematopoietic differentiation, is deleted in acute lymphoblastic leukemia (ALL) (Sun et al., 1999) and has previously been identified as a CIS in a screen conducted in transgenic mice expressing the Notch1 intracellular domain (Notch^IC^) (Beverly and Capobianco, 2003). *Myc* and *Nmyc1* insertions are mutually exclusive with each other and both loci are mutually exclusive with *Notch1*. These three loci may be functionally redundant because *Myc* and *Nmyc1* are related in sequence and function, and *Notch1* insertions can activate *Myc* expression (Sharma et al., 2007).

We also find a rationale within the literature for some of our novel interactions such as the comutation of *Notch1* and *Lfng*. Lfng modulates the activity of the Notch1 receptor by glycosylation and frucosylation of the N-terminal extracellular EGF domains (Stirewalt and Radich, 2003). This suggests that in tumors where *Notch1* is activated *Lfng* insertions can cooperate to enhance its activity.

Complete lists of interacting loci for 5 kb, 30 kb and 300 kb CISs with p values < 0.05 are included (Tables S7, S8, and S6, respectively). As expected for lower kernel widths, we find some adjacent CISs that may affect the same gene are mutually exclusive. For instance, some of the CISs near *Myc* using a 5 kb or 30 kb scale (Figures S5A and S5B) are mutually exclusive events. This may suggest that once an insert is obtained within a CIS there is apparently no selection for insertion within an adjacent CIS affecting the same gene, since this would be a redundant duplication of the same oncogenic function.

### Separate Clusters of Insertions Create Distinct Flt3 and Notch1 Mutants

**Figure 4:**
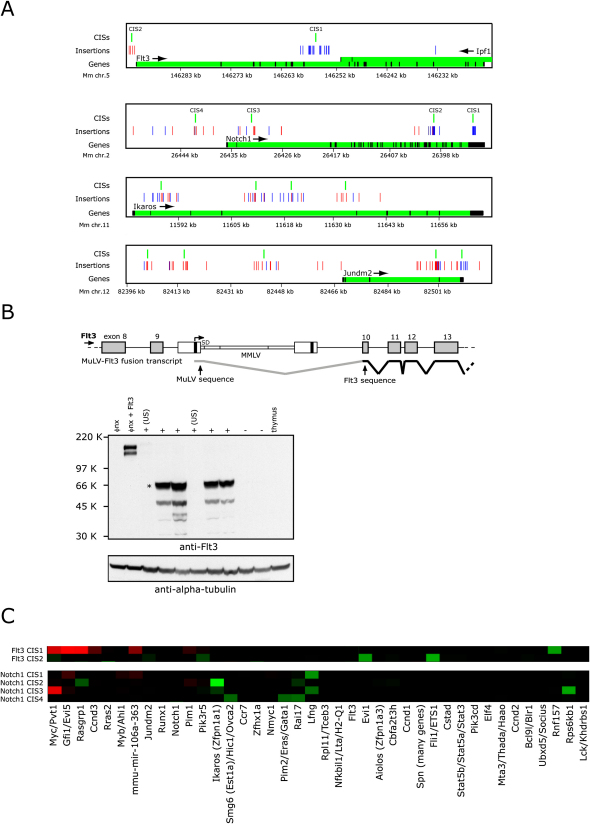
Clusters of Insertions within *Flt3* and *Notch1* Induce Oncogenic Proteins with Distinct Properties (A) Analysis of *Flt3*, *Notch1*, *Ikaros* and *Jundm2* with a 5 kb kernel size reveals multiple CISs. Blue bars represent sense insertions, red bars antisense insertions, green bars introns and black bars exons. (B) Chimeric transcripts of MuLV and *Flt3* sequences are formed by splicing of MuLV splice donor (SD) to splice acceptor of *Flt3* exon10 producing a 1682nt mutant transcript (upper panel), and expression of truncated Flt3 proteins in tumors containing fusion transcripts. Translation starts from an in-frame start codon in exon10 (lower panel). Protein lysates of tumors with (+) or without (−) *Flt3* insertion were separated with SDS/Page followed by immunoblotting with the indicated antibodies. Tumors with insertions upstream of *Flt3* (US). (C) 300 kb CISs co-occurring or mutually exclusive with the separate 5 kb CISs found in *Notch1* and *Flt3*. 5 kb CIS names are indicated on vertical axis, 300 kb CIS names on horizontal axis.

Using a kernel width of 5 kb, we found that insertions in frequently mutated genes like *Flt3*, *Notch1*, *Jundm2* and *Ikaros* are unevenly distributed into clusters that may mutate the same genes by different mechanisms (Figure 4A). Two CISs were found in or near *Flt3*, a gene frequently mutated in human hematopoietic malignancies (Stirewalt and Radich, 2003). One CIS (CIS2^5 kb^) upstream of the gene has 4 insertions where the retroviral genome is inserted in the antisense direction relative to the *Flt3* transcript, suggesting that they act by an enhancer effect on the *Flt3* promoter. The other CIS (CIS1^5 kb^) resides in *Flt3* intron 9–10. In tumors with CIS1^5 kb^ insertions, RT-PCR identified chimeric transcripts that fuse the MuLV transcript to *Flt3* coding sequences and encode an N-terminally truncated protein of approximately 65 kDa (Figure 4B and data not shown). Western blot analysis showed high levels of a mutant Flt3 protein of 65kd in tumors having an insertion in *Flt3* intron 9-10 but not in tumors with insertions upstream of *Flt3* or normal thymus tissue. We also found that tumors with insertions upstream of *Flt3* (CIS2^5 kb^) are significantly enriched for insertions near *Evi1* and *Ets1,* but this is not the case for tumors with *Flt3* truncating insertions (CIS1^5 kb^) (Figure 4C). Rather, truncated *Flt3* is mutually exclusive with mutation of *Myc*, *Gfi1*, and *Rasgrp1*, whereas there is no selection against mutation of these genes in tumors with upstream *Flt3* insertions.

Similarly, CISs found within and upstream of *Notch1* suggest that *Notch1* may also give rise to functionally distinct oncogenic mutants (Figure 4A). CIS4^5 kb^, upstream of *Notch1*, most likely enhances Notch1 expression, whereas CIS3^5 kb^ in intron 2 may induce overexpression of full length Notch1 or mutant Notch1 proteins that lack approximately 50 N-terminal amino acids. The other CISs in *Notch1* give rise to a constitutively active mutant protein consisting of the intracellular domain of Notch1 (Notch^IC^) (CIS2^5 kb^), or remove the destabilizing COOH-terminal PEST-domain and thereby increase Notch1 activity (CIS1^5 kb^) (Hoemann et al., 2000; Weng et al., 2004). Both Notch^IC^ and Notch1 lacking the PEST-domain closely resemble NOTCH1 mutants found in human cancers (Weng et al., 2004). Interestingly, Notch^IC^ (CIS2^5 kb^) mutations are almost exclusively found in wild-type tumors (wild-type versus *p19^ARF−/−^*, 11/3, p value = 0.006, wild-type versus *p53^−/−^*, 11/2, p value = 0.011) indicating that expression of the Notch1^IC^ mutant may be particularly oncogenic in the wild-type background. Moreover, mutation of *Ikaros* is strongly selected for in tumors that have a *Notch^IC^* mutation (p = 7.6 × 10^−5^) but no significant co-occurrence is found with other *Notch1* mutations. Lastly, in contrast to CIS1^5 kb^ and CIS3^5 kb^, mutations in CIS2^5 kb^ (Notch^IC^) do not co-occur with *Lfng* mutations, most likely because Notch^IC^ does not contain the N-terminal extracellular EGF domains and therefore will not be activated by increased Lfng levels (Figure 4C). Together, these data illustrate that increasing the coverage of insertional mutagenesis screens and analysis with a range of kernel sizes is informative even for CIS genes that have previously been established as cancer genes.

### Using Intragenic Insertions to Identify Tumor Suppressor Genes

In some cases, insertions are selected for because they disrupt and inactivate tumor suppressor genes (Suzuki et al., 2006; reviewed in Uren et al., 2005). 4700 of our 10,806 inserts land within the transcribed regions of genes (Table S9). Some of these mutations are within known oncogenes where truncating or enhancer mutations within the gene are oncogenic (as previously discussed for *Flt3* and *Notch1*). However, we also find known tumor suppressor loci.

The most prevalent known tumor suppressor on this list is *Ikaros* (*Zfpn1a1*), harboring 50 insertions. Another family member, *Zfpn1a3* (*Aiolos*), harbors 11 insertions and like *Ikaros* is also implicated in ALL (Mullighan et al., 2007). Other known tumor suppressor loci also carry disrupting insertions including *NfI* (20 insertions), *Ovca2* (6) and *Wwox* (7). Candidates of particular note are: *E2f2* (which can act as a haploinsufficient tumor suppressor of *Myc*-induced lymphoma in mice (Opavsky et al., 2007)), *Raptor* (a binding partner and inhibitor of mTOR (target of rapamycin) (Kim et al., 2002; Hara et al., 2002)), *Nfatc3* (found in a previous retroviral screen to suppress SL3-3 induced lymphoma (Glud et al., 2005) and also found to suppress mammary adenocarcinoma (Lee et al., 2005)), *Xrcc6 (Ku70*) (knockouts of which develop thymic and disseminated T cell lymphomas [Li et al., 1998]) and *Ablim1*, which is located in a chromosomal region frequently lost in human tumors (Kim et al., 1997)(Figure S6). Notably, we observe few inserts within commonly mutated tumor suppressors such as *Cdkn2a*, *Pten* and *Rb1*. These genes may be poor targets for integration due to insertion site preferences of the virus or inability of the virus to inactivate these genes by insertion.

We further postulated that finding multiple intragenic insertions within both alleles of a gene is more likely for tumor suppressor genes than oncogenes. 55 genes carry more than one intragenic insertion within the same tumor (Table 2). To estimate the significance of such events, we compared how often genes were hit twice in real data versus 100,000 permutations of randomized data (shuffling inserts between tumors) and ranked them by this p value.

*Ikaros (Zfpn1a1)* is disrupted in 33 tumors, 11 of which carry more than one intragenic insertion. After shuffling, we find on average only 3 tumors carry more than one insertion within *Ikaros* (p value = 1 x 10^−5^). By comparison, the known oncogene *CyclinD3* (with 148 inserts) is hit more than once in 25 tumors, however this number is similar to that expected by chance (in randomized data 22 tumors have more than one hit, yielding a p value of 0.2). Other known or candidate tumor suppressors with more hits per tumor than expected are indicated in Table 2, including *Mobkl2a* (*hMOB1*), *Ablim1*, *Adrbk1* and *Nf1* (p values 0.02, 0.03, 0.04, 0.08, respectively). We cannot rule out that there may be selection for multiple insertions within the same gene in a single tumor clone even when these insertions are activating mutations. Nonetheless the most established oncogenes of this list (*AhiI*, *Evi1*, *Pim1*, *Flt3*, *Gfi1*, *Evi5*, *Notch1*, and *Ccnd3*) are those with higher p values. Thus, it appears that genes with lower p values in this analysis are better tumor suppressor candidates than those with higher p values.

### Identifying Candidate Cancer Genes by Comparison of Human Tumor Amplicons and Deletions with Murine Retroviral Insertions

**Figure 5:**
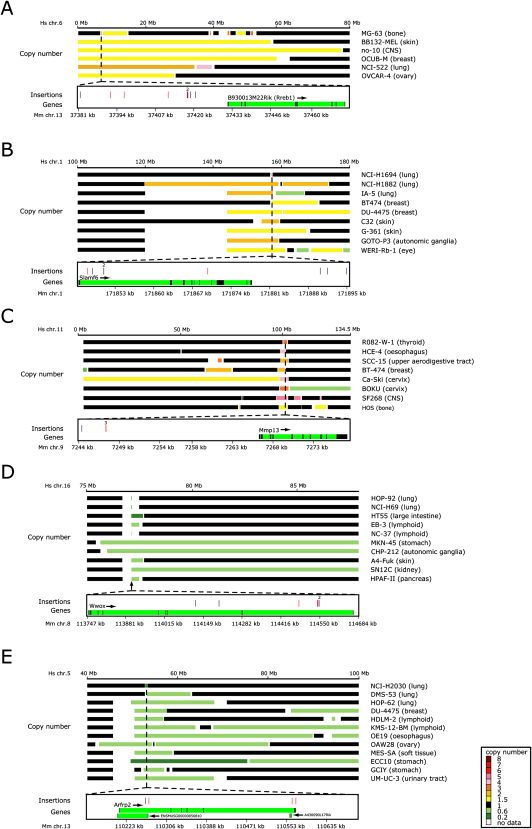
Insertions Identify Tumor Suppressors and Oncogenes in Human Cancers (A–C) *Rreb1* (A), *Slamf6* (B), and *Mmp13* (C) are mutated by multiple insertions and also frequently found amplified in human cancers. (D and E) Mapping of retroviral insertions in the murine *Wwox* (D) and *Arfrp2* (E) genes to deletions found in human cancer cell lines. Upper part, copy number of chromosomal regions in the human cell lines is depicted in color. Names of human cell lines and tissue of origin are provided. Lower part, insertions in murine tumors. Blue bars represent insertions in sense orientation, red bars antisense insertions, green bars introns and black bars exons. Positions on the murine and human chromosomes are indicated on the black horizontal bars in kb and Mb, respectively.

As part of the Wellcome Trust Sanger Institute's Cancer Genome Project, 713 human cell lines were hybridized to 10,000 probe SNP arrays and copy number information was extracted from these data. Some amplicons and deletions span megabases and contain many genes, thus making cancer gene identification difficult. To this end, we mapped our CIS loci to their orthologous loci in the human genome and looked for overlap with amplicons and deletions in the tumor cell lines. The human orthologs of our list of CIS candidate target genes were found to be amplified at a significantly higher frequency than a random list of genes (p value = 0.03). Significant results were also obtained using the list of genes nearest to the midpoint of our CISs (p value = 0.006). Known oncogenes like *Fgfr2*, *Kit* and *Evi1* and other notable examples identified in this manner are illustrated in Figure S7 and Figure 5.

A recurrent amplicon on chromosome 6 is orthologous to a CIS in the vicinity of *Rreb1* (Figure 5A). Rreb1 binds and represses expression of the *p16(Ink4a)* promoter, and the development of pristane-induced plasma cell tumors in Balb/C mice is attributable to a polymorphism in this Rreb1 binding site (Zhang et al., 2003).

Another amplified region on chromosome 1 contains at least 21 genes, of which *Slamf6* appears to be the most likely target gene of our insertions (Figure 5B). Polymorphisms within the region of *Slamf6* (Ly108) have been implicated in systemic lupus erythematosus (SLE) (Wandstrat et al., 2004).

A recurrent amplicon on Chromosome 11 contains at least 24 genes, including a cluster of genes encoding matrix metalloproteinases (MMPs). Several MMPs within this amplicon have been previously implicated in cancer, however the only one of these genes implicated by our insertions is *Mmp13*, which to date has not been ascribed a role in cancer (Figure 5C).

We did not see significant global overlap with our lists of tumor suppressor candidate genes and the deletions of the human CGH data. This is perhaps not surprising since, while these lists may be enriched for tumor suppressor genes, they still contain many oncogenes that have insertions within their transcribed regions. Nonetheless, we also find candidate tumor suppressor genes that overlap recurrent deletions. *Wwox* is disrupted by seven intragenic insertions and deleted within 10 of the human cell lines (Figure 5D). A novel candidate tumor suppressor emerging from this comparison is *Arfrp2* (*Arl15*)(Figure 5E). *Arfrp2* is a member of the ADP-ribosylation factor-like family. Notably, another member of this family, *ARL11* (*ARLTS1*), is a tumor suppressor gene where truncating germline mutations or promoter methylation contribute to leukemia, breast cancer, ovarian cancer, and melanoma (Sych et al., 1978; Frank et al., 2005; Petrocca et al., 2006).

## Discussion

Here, we report on a large-scale retroviral insertional mutagenesis screen using more than 500 tumors from *p19^ARF−/−^*, *p53^−/−^* and wild-type mice. This scale of analysis allows identification of a high number of new candidate oncogenes and tumor suppressors and detects highly significant combinations of co-occurring or mutually exclusive genes. Notably, a significant proportion of CISs that map to orthologs of known human cancer loci are not only identified in hematopoietic tumors, but also in other tumor types such as lung, colon, breast, and prostate tumors.

We have identified 25 CISs that were significantly more mutated in one of the germline genotypes compared to either one or both of the two other genotypes. Together with *p53* and *RAS*, *FLT3* is the most commonly mutated gene in human AML, occurring in approximately 25% of cases (Stirewalt and Radich, 2003). *Flt3* is almost exclusively mutated in *p53^−/−^* and *p19^ARF−/−^* tumors and not in wild-type tumors (11, 13 and 2 insertions, respectively), indicating that *Flt3* mutations may be particularly oncogenic in the absence of a functional p19^ARF^-MDM2-p53 pathway. Flt3 induces Ras signaling (Stirewalt and Radich, 2003), and mutation of *Flt3* is mutually exclusive with *Ras* mutations in human cancers suggesting that mutation of *Flt3* has similar effects as *Ras* mutation (Stirewalt et al., 2001). We find that mutations in the Ras-activating *Rasgrp1* (Ebinu et al., 2000) are mutually exclusive with mutation of *Flt3*, also suggesting that activation of Ras signaling may be an important effect of *Flt3* mutation in tumors.

The utility of retroviral insertions is further illustrated by comparison to array CGH data from cancer cell lines. We identify several novel candidate cancer genes including *Rreb1*, *Mmp13* and *Arfrp2* (*Arl15*). Similarly useful comparisons can also be envisaged for tumor resequencing data. Human populations carry many polymorphisms, only some of which contribute to tumor susceptibility and many tumors have a mutator phenotype that creates a background of irrelevant mutations. In such studies polymorphisms and background mutations can only be distinguished from oncogenic mutations by stringent statistics that may inadvertently exclude rare but genuine oncogenic events. CIS loci should be a useful tool to help focus future resequencing studies, as illustrated by a recent study which finds overlap between mutations in coding regions of human breast cancer genes and CIS loci from an MMTV insertional mutagenesis study (Theodorou et al., 2007; Wood et al., 2007). As such, CIS associations could be used to prioritize which rarely mutated genes should be sequenced within a tumor set i.e., identifying the commonly mutated genes in a set of tumors and then sequencing their known collaborators from insertional mutagenesis screens.

Due to the tropism of MuLV, our screen is limited in its ability to identify cancer genes from nonhematopoietic tumors. As the technology of transposon mutagenesis matures a greater range of tumor types will become amenable to analysis by insertional mutagenesis. New models notwithstanding, there is still much to be gained from traditional MuLV screens. Larger numbers of inserts and better estimates of tumor clonality will improve the power of association studies, giving rise to interaction maps that are denser and more informative. Even if complete saturating coverage of each tumor is possible, it remains unclear how many more loci might be identified by increasing the number of tumors analyzed. Certainly it appears that screening a greater variety of genotypes and predisposing mutations will expand the range of mutations found. Also, given the differences in susceptibility for tumor development between different inbred mouse strains, it would be interesting to compare the spectrum of oncogenic mutations between these strains. Analysis of MuLV tumors by expression arrays and by linker-mediated PCR on cDNA of all tumors will increase the accuracy with which target genes of insertions can be identified. CGH copy number arrays and ORF resequencing might also identify cooperating mutations that are less amenable to mutation by insertion.

Ultimately there may also be clinical applications of mutually exclusive interactions of CIS loci. Recent studies have indicated that *EGFR* mutations are mutually exclusive with *KRAS* mutations in lung adenocarcinomas (Pao et al., 2005). Mutant *EGFR* is a target for gefitinib and erlotinib, and patients with mutations in *KRAS* instead of *EGFR* do not see any benefit from the use of these drugs. In cases where the target of a drug is unknown or unclear, knowing the associations of nontarget mutations that correlate with treatment outcomes can inform the search for the actual target and/or suggest novel indications for established therapies.

## Experimental Procedures

### MuLV Infection of *p19^ARF−/−^*, *p53^−/−^*, and Wild-Type Mice

Crosses between F0 parental FVB mice +/− for the *p19^ARF^* knockout allele (Kamijo et al., 1997) were performed to generate F1 *p19^ARF−/−^*, *p19^ARF+/−^* and *p19^ARF+/+^* (wild-type) offspring. F0 parental FVB mice^+/−^ for the *p53* knockout allele (Donehower et al., 1992) were crossed to generate *p53^−/−^*, *p53^+/−^* and *p53^+/+^* F1 offspring. Newborn F1 pups were injected i.p. with 1.10^5^ infectious units of MuLV. Animals were monitored in time for the development of tumors, moribund mice were sacrificed and tumors were isolated. All animal experiments were done conform national regulatory standards approved by the DEC (Animal Experiments Committee).

### Identification of Retroviral Insertion Sites

Genomic DNA was isolated using PureGene from Gentra Systems, Inc. Insertions sites were identified using an linker-mediated PCR protocol adapted from Mikkers et al. (Mikkers et al., 2002). PCR products were shotgun subcloned using a protocol developed at the Wellcome Trust Sanger Institute (both protocols available on http://mutapedia.nki.nl/).

### Mapping of Insertions

Detailed instructions and parameters used for informatics are available on request. Briefly, we used cross_match (Dr. Philip Green, unpublished data) to identify vector, primer, linker and viral U5 LTR sequences in the reads. We used SSAHA2 (Ning et al., 2001) to map individual reads onto the mouse genome (NIH Build 34). Chimeric and concatameric subcloning products were separated where possible or else discarded. Sequences containing a splinkerette but lacking an LTR were discarded, unless they were within 2000 bp of contigs that containing an LTR-genome junction, in which case they were added to these contigs where orientation was consistent. A final round of filtering to remove PCR artifacts was then applied.

### Software

Pathway analysis was performed using Ingenuity Pathway Analysis software (Ingenuity® Systems, www.ingenuity.com). Cytoscape® software (version 2.4.0, www.cytoscape.org) was used to create the CIS interaction network.

### Identification of CISs Near Orthologs of Human Cancer Genes

Each of the 30 kb CISs was examined for the presence of a murine ortholog of a human cancer gene within 200 kb up and downstream of the CIS peak. The number of unique orthologs was counted. Next, the number of insertions in the dataset was decreased step-wise to zero by removing insertions from 50 randomly selected mice. CIS positions are determined each time 50 mice are taken out of the dataset, as well as the number of unique orthologs. The experiment was repeated 20 times, average and standard deviation of the number of murine orthologs was determined. To reduce computation time required for the saturation analysis, CISs were determined using an alternative method that applies a more stringent α-level then GKC to determine CISs and therefore detects less CISs.

### Statistical Methods

Significance of genotype-CIS interactions and CIS-CIS interactions were estimated by permutation. Briefly, inserts were placed within a 2 × 2 contingency table based on whether the insert is found within a given CIS and either the genotype of the mouse or the presence or absence of insertions from another CIS within the same mouse. Similar tables were constructed for 100,000 random permutations of data where the entire set of insertions was shuffled between all tumors. A Chi squared test statistic was calculated for each of the real and permuted tables. p values were calculated as the proportion of permuted test statistics that are greater than the real test statistic.

### CGH Comparison

We obtained 10K SNP array CGH data for 713 human cancer cell lines from the Wellcome Trust Sanger Institute (ftp://ftp.sanger.ac.uk/pub/cgp/10kdata). We identified regions of copy number change in each cell line and identified human orthologs of mouse CIS candidate genes overlapping with amplicons and deletions in the human cancer cell lines. For further details see Supplemental Experimental Procedures.

### Transfection and Plasmids

Phoenix cells were transfected using CaPO_4_ precipitation. The LXSN-Flt3 vector used as a positive control for Flt3 expression was obtained from Dr. Olivier Rosnet, Inserm, Marseille, France.

### Protein Extraction and Western Blotting

Protein was isolated from frozen tumor material or Phoenix cells and loaded on SDS-PAGE gels. Antibodies used are Flt3 (8F2) (Cell Signaling Technology, Inc) and alpha tubulin (Sigma-Aldrich, Inc). A protein lysate from a thymus isolated from a 40-day-old wild-type mouse was used as control.

## Figures and Tables

**Table 1:** Genotype-Specific 30 kb CISs

Candidate Gene	CIS Position chr, bp	p53^−/−^ versus WT (P Value)	p53^−/−^ versus p19^ARF−/−^ (P Value)	p19^ARF−/−^ versus WT (P Value)	p19^ARF−/−^ versus p53^−/−^ (P Value)	WT versus p19^ARF−/−^ (P Value)	WT versus p53^−/−^ (P Value)
Ccnd3	17, 45047722	53/19 (0.00003)	−	59/19 (0.00239)	−	−	−
Flt3	5, 146256166	10/0 (0.0009)	−	10/0 (0.0074)	−	−	−
mmu-mir-106a-363	20, 47271889	35/14 (0.00202)	−	40/14 (0.02233)	−	−	−
Runx1 CIS1	16, 91913533	9/0 (0.00185)	−	14/0 (0.00129)	−	−	−
Runx1 CIS5	16, 92317511	10/1 (0.00536)	−	−	−	−	−
Gfi1b	2, 28536129	7/0 (0.00665)	−	−	−	−	−
Pim2	20, 6107503	−	9/2 (0.00868)	−	−	−	−
Ssbp3/Thea CIS1	4, 105792860	−	4/0 (0.02532)	−	−	−	−
Rps6kb1	11, 86313637	−	4/0 (0.02355)	−	−	−	−
Chc1l	14, 67474050	−	4/0 (0.02368)	−	−	−	−
Sp1	15, 102462245	−	4/0 (0.02445)	−	−	−	−
Smg6 (Est1a)	11, 74741154	−	−	19/3 (0.00882)	19/1 (0.00088)	−	−
Zhfx1a	18, 5356615	−	−	14/2 (0.02181)	−	−	−
Eras/Gata1	20, 6188924	−	−	8/0 (0.02518)	−	−	−
Notch1 CIS2	2, 26433345	−	−	−	13/1 (0.01197)	−	−
Ikaros (Zfpn1a1)	11, 11611278	−	−	−	11/1 (0.02373)	−	14/1 (0.00095)
Pim1 CIS1	17, 27209067	−	−	−	11/1 (0.02365)	−	−
Notch1 CIS1	2, 26396468	−	−	−	−	21/11 (0.0053)	21/6 (0.00553)
Gfi1 CIS2	5, 106805956	−	−	−	−	72/66 (0.01741)	−
Ptma	1, 86244930	−	−	−	−	4/0 (0.02583)	−
Phf10/Tcte3	17, 12963705	−	−	−	−	4/0 (0.02577)	−

Contingency tables were used to calculate whether CISs were preferentially mutated in any of the three genotypes compared to the other genotypes. The number of insertions in the CIS for the respective genotypes is indicated. p values are between brackets.

**Table 2:** Genes with Multiple Intragenic Insertions

Gene Name (Other Names)	# Inserts Total	# Tumors Hit Total	# Tumors Hit > 1	Expected # Tumors Hit > 1	P Value
Anks1	2	1	1	0	0
Myo1g	2	1	1	0	0
Nr1d1	2	1	1	0	0
Zfpn1a1 (Ikaros)	50	33	11	3.04	1.00E-05
1300010F03Rik	3	2	1	0.01	0.01
2700049A03Rik	3	2	1	0.01	0.01
Smg6 (Est1a)	35	29	5	1.53	0.01
Antxr1	3	2	1	0.01	0.01
Btk	3	2	1	0.01	0.01
Ccr7	13	11	2	0.21	0.01
Cnot2	3	2	1	0.01	0.01
Fgfr1	3	2	1	0.01	0.01
Nedd9	3	2	1	0.01	0.01
Nxf1	3	1	1	0.01	0.01
Rps6kb2	3	2	1	0.01	0.01
Trim30	3	2	1	0.01	0.01
4921504K03Rik'(Iqch)	4	3	1	0.02	0.02
5330417K06Rik'(Mobkl2a/hMOB1)	4	3	1	0.02	0.02
Foxp1	4	3	1	0.02	0.02
Rbm10	4	3	1	0.02	0.02
Ablim1	5	4	1	0.03	0.03
Glb1	5	4	1	0.03	0.03
Sema4b	5	4	1	0.03	0.03
Adrbk1	6	5	1	0.04	0.04
Nfatc1	6	5	1	0.04	0.04
ENSMUSG00000066549	6	5	1	0.04	0.04
ENSMUSG00000068244	6	5	1	0.04	0.04
Prg1	6	5	1	0.04	0.04
Rai17	17	15	2	0.36	0.04
ENSMUSG00000050227	27	24	3	0.92	0.05
Ifi47	7	6	1	0.06	0.06
ENSMUSG00000069613	7	6	1	0.06	0.06
Padi2	7	6	1	0.06	0.06
Rasgrp1	7	6	1	0.06	0.06
Evi2b	8	7	1	0.08	0.07
Runx1	51	45	6	3.17	0.07
Tgtp	8	7	1	0.07	0.07
Bcl2l1	8	6	1	0.08	0.08
Nf1	8	7	1	0.08	0.08
Ubxd5 (Socius/Otud5)	10	9	1	0.12	0.12
Pecam1	10	9	1	0.12	0.12
XP_126601.5 (Slc16a5/MCT6)	11	10	1	0.15	0.14
Zfpn1a3 (Aiolos)	11	10	1	0.15	0.14
DXImx46e	12	11	1	0.18	0.17
Ccnd3	148	112	25	22.05	0.22
Notch1	47	41	4	2.7	0.28
Jundm2	30	27	2	1.13	0.31
Rpl11	17	16	1	0.36	0.31
2610009E16Rik'(Ptms)	17	16	1	0.36	0.32
Evi5	50	46	4	3.05	0.36
Gfi1	19	18	1	0.46	0.38
Flt3	21	20	1	0.55	0.44
Pim1	21	20	1	0.56	0.44
Evi1	24	23	1	0.72	0.54
Ahi1	31	30	1	1.2	0.73

The p value of genes being mutated by two intragenic insertions in the tumors was estimated by comparison to 100,000 permutations of randomized data.

## Supplemental Data

Document S1. Seven Figures and Six Tables
