# Supplementary material for: Large-Scale Mutagenesis in p19ARF- and p53-Deficient Mice Identifies Cancer Genes and Their Collaborative Networks
Source: Cell. 2008 May 16;133(4):727–41. doi: 10.1016/j.cell.2008.03.021 (PMC2405818; doi:10.1016/j.cell.2008.03.021)
Supplement: Document S1. Seven Figures and Six Tables [file mmc1.pdf]

Cell, Volume *133*

Supplemental Data

Large-Scale Mutagenesis in *p19*^{ARF}- and *p53*-Deficient Mice Identifies Cancer Genes and Their Collaborative Networks

Anthony G. Uren, Jaap Kool, Konstantin Matentzoglu, Jeroen de Ridder, Jenny Mattison, Miranda van Uitert, Wendy Lagcher, Daoud Sie, Ellen Tanger, Tony Cox, Marcel Reinders, Tim J. Hubbard, Jane Rogers, Jos Jonkers, Lodewyk Wessels, David J. Adams, Maarten van Lohuizen, and Anton Berns

Figure S1

A

	Spleen			Thymus			Lymph nodes		
	%CD3	%B220	number of tumors	%CD3	%B220	number of tumors	%CD3	%B220	number of tumors
p19$^{ARF-/-}$ (n=167)	56	18	92	67	3	65	22	63	10
p53$^{-/-}$ (n=47)	33	22	31	78	2	15	8	25	1
wt (n=135)	51	14	83	43	4	47	47	30	5

B

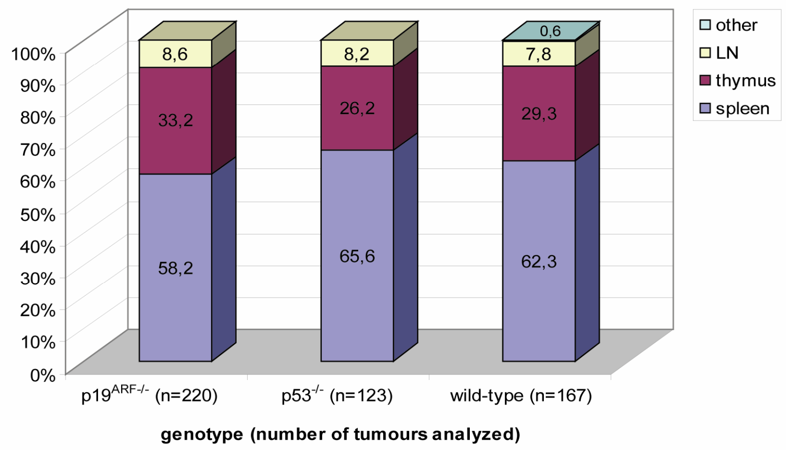

Figure S1. T-cell and B-cell content and tissue of origin of analyzed tumors. (A) Average T-cell and B-cell content per tissue per genotype of the analyzed tumors as determined by flow cytometry using antibodies for CD3ε and CD45R/B220 (BD Biosciences). (B) Tissue of origin of the analyzed tumors. For each genotype, the proportion of the tumors isolated from spleen, thymus and lymph nodes and other organs is depicted.

Figure S2

A

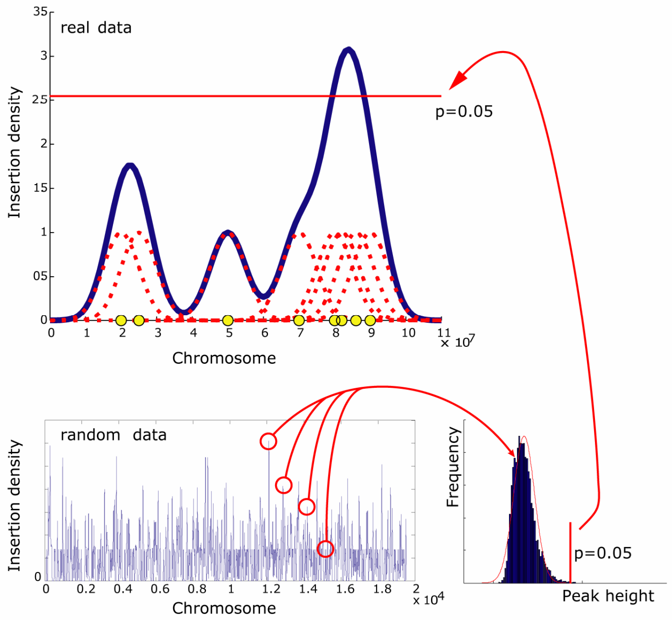

B

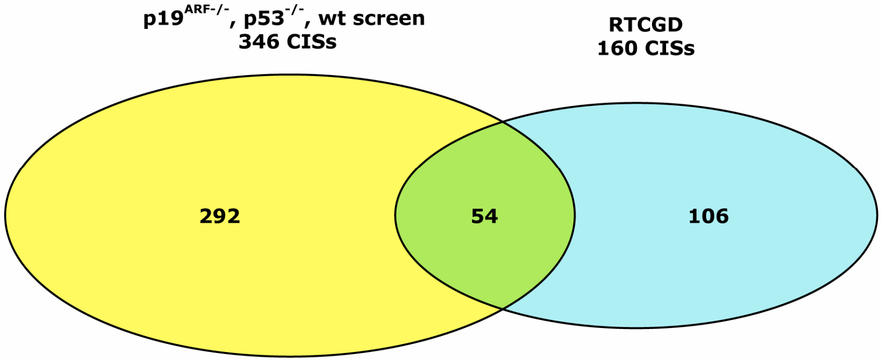

Figure S2

Figure S2. The Gaussian kernel convolution method and comparison to RTCGD data. The Gaussian kernel convolution method places a kernel over each insertion in the genome, and subsequently measures the insertion density of the real data (upper panel) by convoluting the independent kernels. The real insertion density is compared to a randomized dataset (left lower panel): frequency of insertion densities in random data is measured and used to calculate the significance (p-value) of a local enrichment of insertions (right lower panel). Kernels are indicated with red dotted line, insertions by yellow circles and insertion density with the blue line.

Figure S3

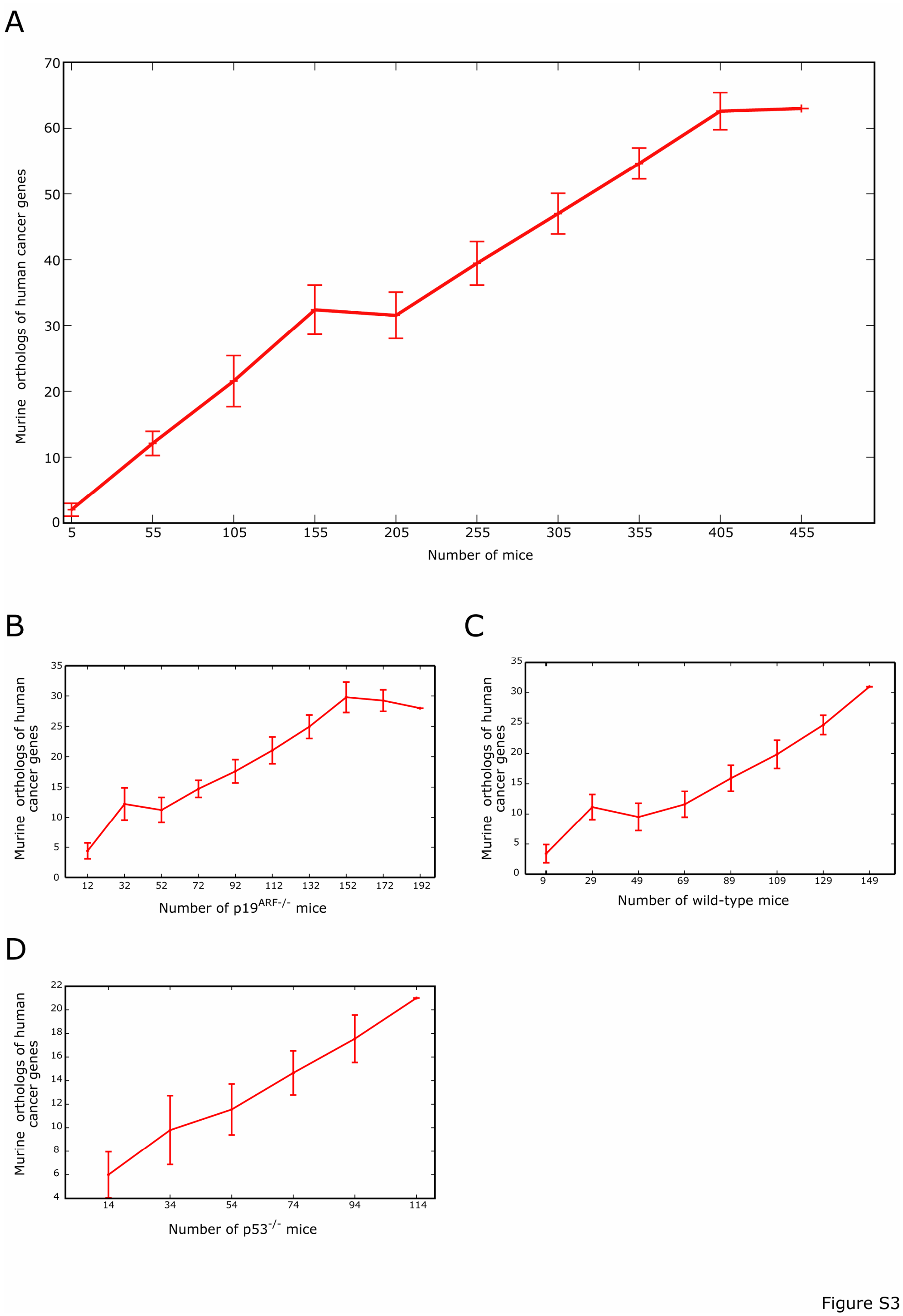

Figure S3

Figure S3. Estimation of the saturation for discovery of cancer genes. The number of CISs found near murine orthologs of known human cancer genes increase with the number of insertions in the dataset. (A) All mice analyzed together. To examine if we reach saturation in any of the individual panels, we performed similar analyses on the cohorts of mice from the three genotypes separately. The results suggest saturation of the $p19^{ARF-/-}$ cohort, but not in $p53^{-/-}$ or wild-type mice. (B) $p19^{ARF-/-}$ mice. (C) Wild-type mice.(D) $p53^{-/-}$ mice. The average of 20 experiments is indicated, vertical bars indicate standard deviation. The data are plotted as the mean $\pm$ the standard deviation.

Figure S4

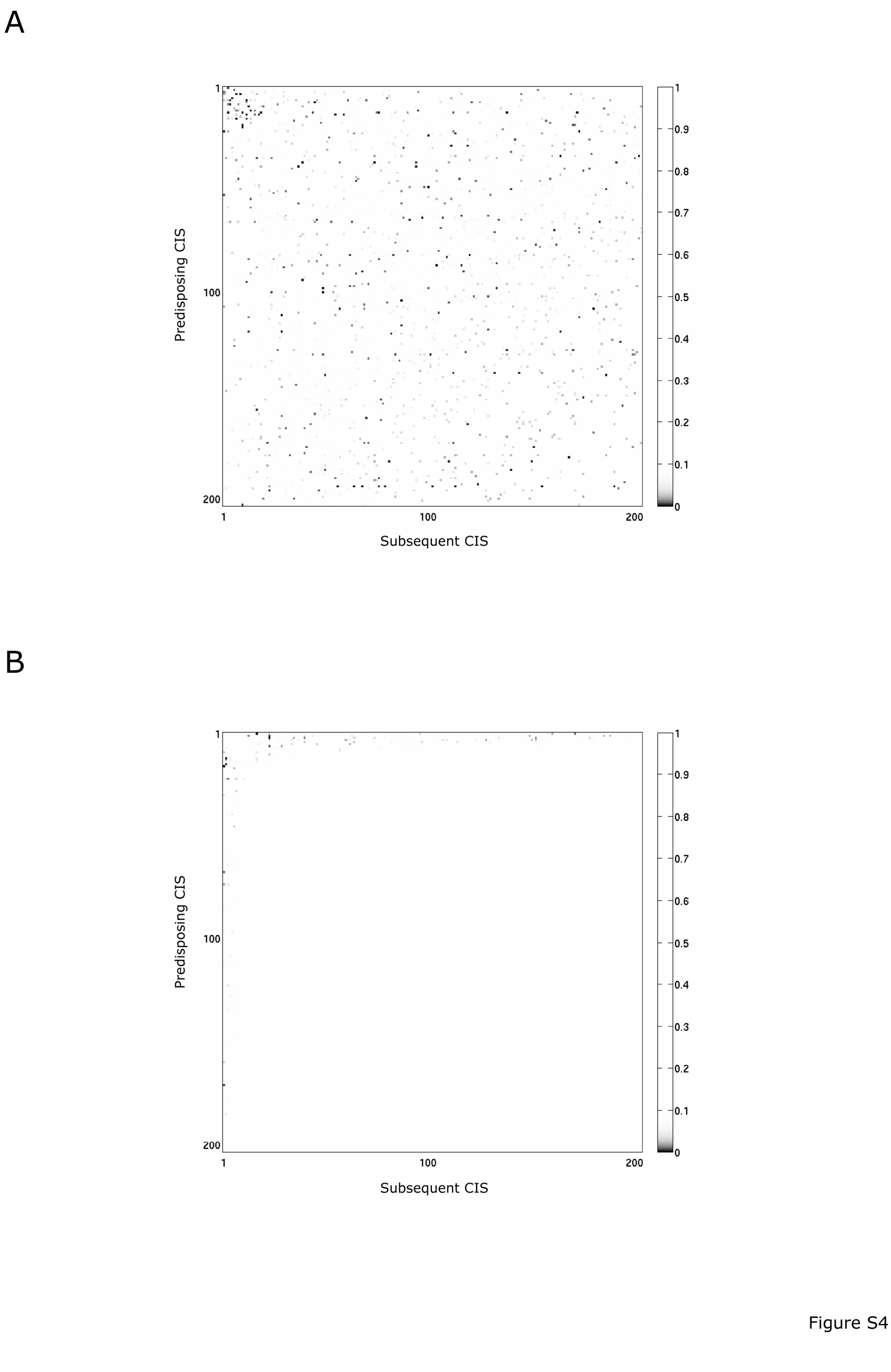
A
Predisposing CIS
Subsequent CIS
1
100
200
0
0.1
0.2
0.3
0.4
0.5
0.6
0.7
0.8
0.9
1
B
Predisposing CIS
Subsequent CIS
1
100
200

Figure S4. Interaction maps of 203 300 kb CISs. Top panel, co-occurrence between 300 kb CISs, lower panel mutual exclusivity between CISs. P-values for interactions are represented in grayscales.

Figure S5

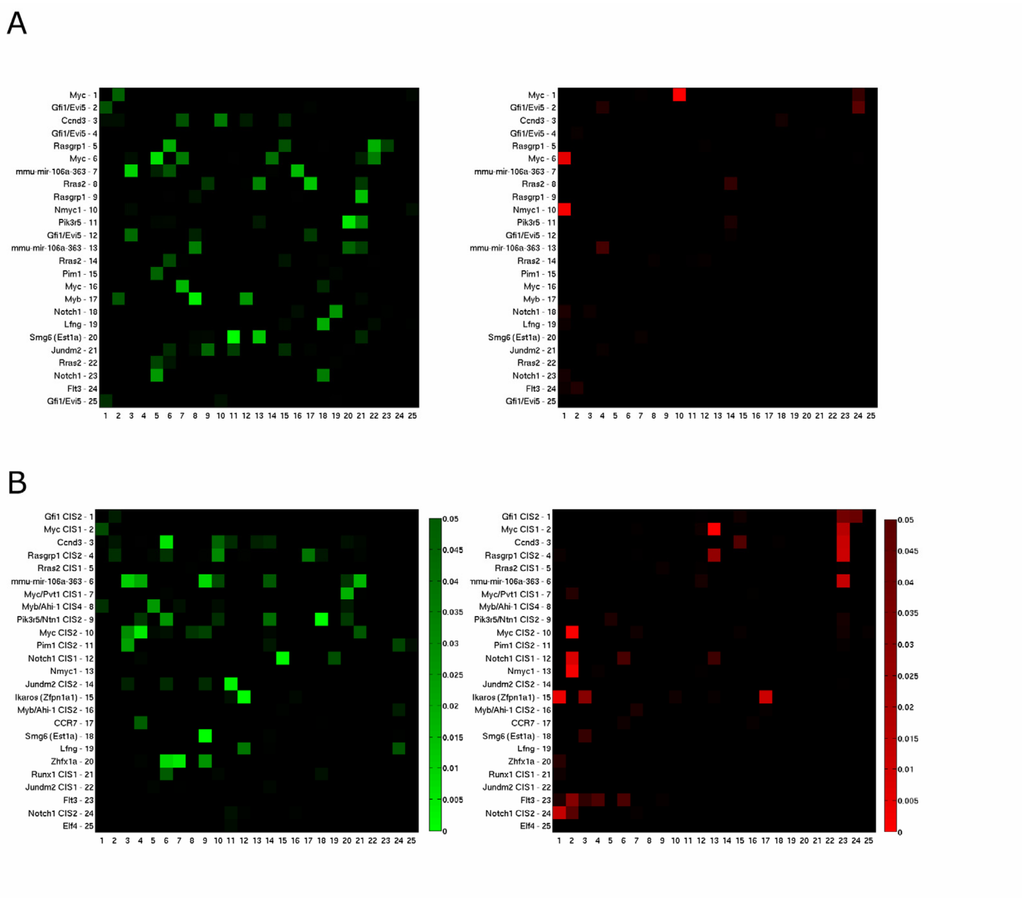
A
Myc - 1
Gfi1/Evi5 - 2
Ccnd3 - 3
Gfi1/Evi5 - 4
Rasgrp1 - 5
Myc - 6
mmu-mir-106a-363 - 7
Rras2 - 8
Rasgrp1 - 9
Nmyc1 - 10
Pik3r5 - 11
Gfi1/Evi5 - 12
mmu-mir-106a-363 - 13
Rras2 - 14
Pim1 - 15
Myc - 16
Myb - 17
Notch1 - 18
Lfng - 19
Smg6 (Est1a) - 20
Jundm2 - 21
Rras2 - 22
Notch1 - 23
Flt3 - 24
Gfi1/Evi5 - 25
1 2 3 4 5 6 7 8 9 10 11 12 13 14 15 16 17 18 19 20 21 22 23 24 25
B
Gfi1 CIS2 - 1
Myc CIS1 - 2
Ccnd3 - 3
Rasgrp1 CIS2 - 4
Rras2 CIS1 - 5
mmu-mir-106a-363 - 6
Myc/Pvt1 CIS1 - 7
Myb/Ahi-1 CIS4 - 8
Pik3r5/Ntn1 CIS2 - 9
Myc CIS2 - 10
Pim1 CIS2 - 11
Notch1 CIS1 - 12
Nmyc1 - 13
Jundm2 CIS2 - 14
Ikaros (Zfpn1a1) - 15
Myb/Ahi-1 CIS2 - 16
CCR7 - 17
Smg6 (Est1a) - 18
Lfng - 19
Zhfx1a - 20
Runx1 CIS1 - 21
Jundm2 CIS1 - 22
Flt3 - 23
Notch1 CIS2 - 24
Elf4 - 25
1 2 3 4 5 6 7 8 9 10 11 12 13 14 15 16 17 18 19 20 21 22 23 24 25
0.05
0.045
0.04
0.035
0.03
0.025
0.02
0.015
0.01
0.005
0

Figure S5. Mapping interaction networks between Common Insertion Sites. (A) Co-occurrence (left) and mutual exclusivity (right) between the top 25 5 kb CISs. CIS names and CIS rank is indicated on vertical axis, numbers on horizontal axis are CIS rank. P-values for interactions are represented in green. The horizontal axis represents CISs that are assumed to be the predisposing, more clonal event and the vertical axis represents CISs that are presumed to be subsequent, subclonal events. (B). Co-occurrence (left) and mutual exclusivity (right) between the top 25 30 kb CISs. Set up of the figure as described in (A).

Figure S6

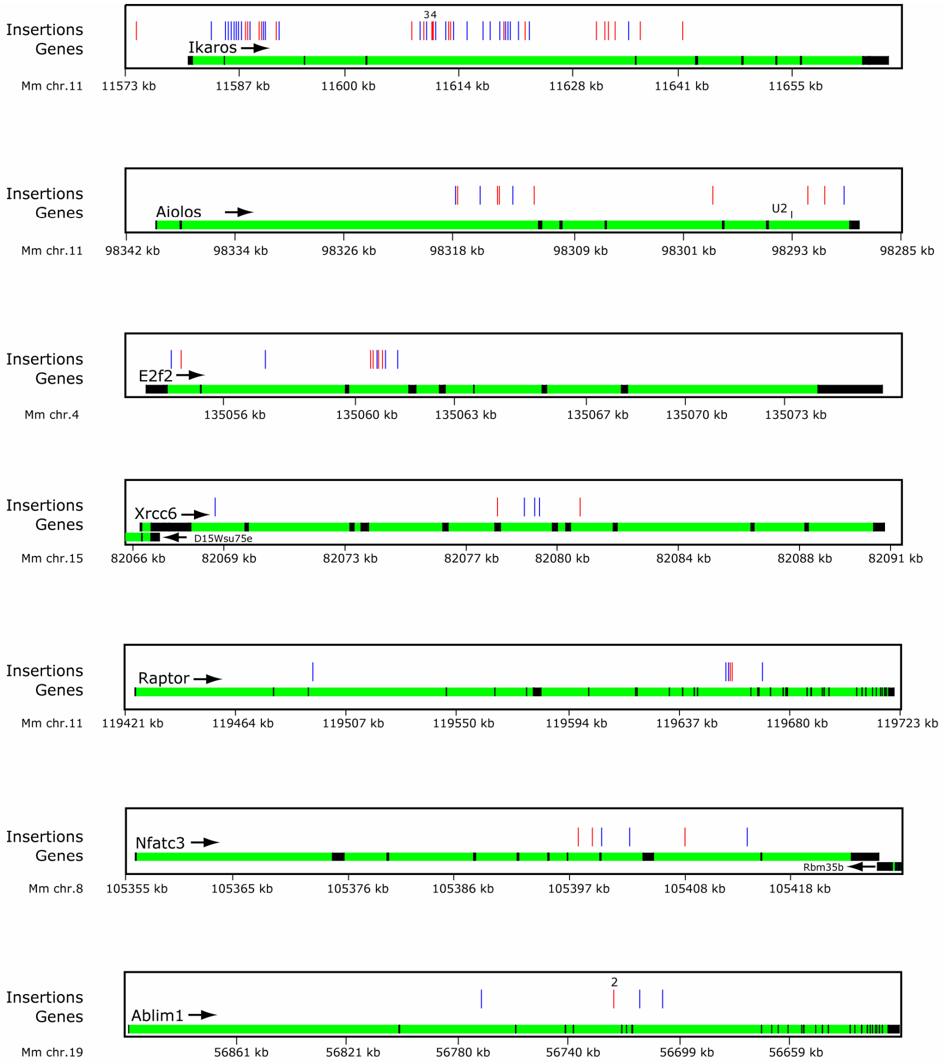

Insertions
Genes
34
Ikaros
Mm chr.11
11573 kb
11587 kb
11600 kb
11614 kb
11628 kb
11641 kb
11655 kb
Insertions
Genes
Aiolos
U2
Mm chr.11
98342 kb
98334 kb
98326 kb
98318 kb
98309 kb
98301 kb
98293 kb
98285 kb
Insertions
Genes
E2f2
Mm chr.4
135056 kb
135060 kb
135063 kb
135067 kb
135070 kb
135073 kb
Insertions
Genes
Xrcc6
D15Wsu75e
Mm chr.15
82066 kb
82069 kb
82073 kb
82077 kb
82080 kb
82084 kb
82088 kb
82091 kb
Insertions
Genes
Raptor
Mm chr.11
119421 kb
119464 kb
119507 kb
119550 kb
119594 kb
119637 kb
119680 kb
119723 kb
Insertions
Genes
Nfatc3
Rbm35b
Mm chr.8
105355 kb
105365 kb
105376 kb
105386 kb
105397 kb
105408 kb
105418 kb
Insertions
Genes
2
Ablim1
Mm chr.19
56861 kb
56821 kb
56780 kb
56740 kb
56699 kb
56659 kb

Figure S6. Insertions within gene identify known and candidate tumor suppressor genes. Examples of genes that have multiple insertions within the open reading frame are depicted. Blue bars represent sense insertions, red bars anti-sense insertions, green bars introns and black bars exons. Numbers above insertions indicate the number of insertions found at that location.

Figure S7

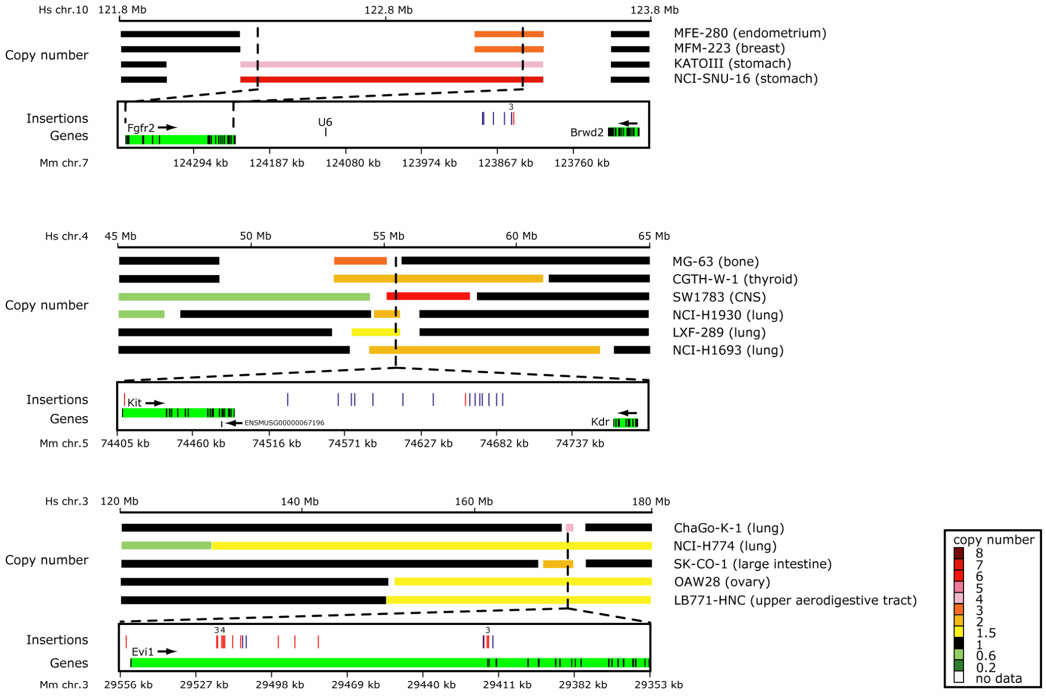

Figure S7

Figure S7 Insertions identify tumor suppressors and oncogenes in human cancers. *Fgfr2*, *Kit*, *Evi1* are mutated by multiple insertions and also frequently found amplified in human cancers. Upper part, copy number of chromosomal regions in the human cell lines is depicted in color. Names of human cell lines and tissue of origin are provided. Lower part, insertions in murine tumors. Blue bars represent insertions in sense orientation, red bars anti-sense insertions, green bars introns and black bars exons. Positions on the murine and human chromosomes are indicated on the black horizontal bars in kb and Mb respectively.

Table S1 Number of CISs dependent on kernel size and p-value

Kernel width (bp)	p = 0.001	p = 0.005	p = 0.01	p = 0.05	p = 0.1
1000	190	208	219	304	393
5000	215	272	305	352	373
10000	263	276	293	347	385
30000	235	258	271	346	377
50000	214	231	258	299	323
100000	180	201	220	262	281
150000	164	182	191	236	254
300000	145	160	175	203	210

Table S2. Identification of 346 30kb CISs and their candidate target genes

The most likely candidate target genes of the CISs were selected on the basis of the orientation and position of the insertions relative to the genes in the vicinity. Known cancer genes (Futreal et al., 2004) are indicated in bold.

Rank	Peak Height	Chr.	Position	CIS name	ENSID of target gene
1	201.74	5	106805956	Gfi1 CIS2	ENSMUSG00000029275
2	189.99	15	61996945	**Myc** CIS1	ENSMUSG00000022346
3	130.35	17	45047722	**Ccnd3**	ENSMUSG00000034165
4	119.99	2	116925138	Rasgrp1 CIS2	ENSMUSG00000027347
5	84.16	7	108013127	Rras2 CIS1	ENSMUSG00000055723
6	82.19	X	47271889	mmu-mir-106a-363	ENSMUSG00000065456
7	54.74	15	62187673	**Myc**/Pvt1 CIS1	ENSMUSG00000022346
8	51.54	10	21169584	Myb/Ahi-1 CIS4	ENSMUSG00000019982
9	49.2	11	68156417	Pik3r5/Ntn1 CIS2	ENSMUSG00000020901
10	47.62	15	62053819	**Myc** CIS2	ENSMUSG00000022346
11	41.16	17	27304632	**Pim1** CIS2	ENSMUSG00000024014
12	37.62	2	26396468	**Notch1** CIS1	ENSMUSG00000026923
13	35	12	12295320	**Nmyc1**	ENSMUSG00000037169
14	34.21	12	82502431	Jundm2 CIS2	ENSMUSG00000034271
15	31.68	11	11611278	Ikaros (Zfpn1a1)	ENSMUSG00000018654
16	29.82	10	21006970	Myb/Ahi-1 CIS2	ENSMUSG00000019982
17	25.02	11	98977657	CCR7	ENSMUSG00000037944
18	24.71	11	74741154	Smg6 (Est1a)	ENSMUSG00000038290
19	24.66	5	139597100	Lfng	ENSMUSG00000029570
20	24.33	18	5356615	Zhfx1a	ENSMUSG00000024238
21	23.39	16	91913533	**Runx1** CIS1	ENSMUSG00000022952
22	22.23	12	82415460	Jundm2 CIS1	ENSMUSG00000034271
23	20.37	5	146256166	**Flt3**	ENSMUSG00000042817
24	19.67	2	26433345	**Notch1** CIS2	ENSMUSG00000026923
25	19.52	X	42948379	Elf4	ENSMUSG00000031103
26	17.84	4	148193835	Pik3cd	ENSMUSG00000039936
27	16.86	16	92317511	**Runx1** CIS5	ENSMUSG00000022952
28	15.8	8	122087873	**Cbfa2t3h**	ENSMUSG00000006362
29	15.54	17	27209067	**Pim1** CIS1	ENSMUSG00000024014
30	13.77	X	6107503	Pim2	ENSMUSG00000031155
31	13.41	3	29513696	**Evi1/Mds1**	ENSMUSG00000027684
32	12.93	16	92355461	**Runx1** CIS6	ENSMUSG00000022952
33	12.52	11	87474817	mmu-mir-142	ENSMUSG00000065420
34	12.32	9	32532718	Ets1/**Fli1**	ENSMUSG00000032035
35	12.27	11	78729767	Nos2/Ksr/Lgals9	ENSMUSG00000020826
36	12.22	11	74899117	Smg6/rno-mir-132&212/Ovca2/Hic1 CIS1	ENSMUSG00000038290
37	12.19	11	75003637	Smg6/rno-mir-132&212/Ovca2/Hic1 CIS2	ENSMUSG00000038290
38	11.62	16	91967594	**Runx1** CIS2	ENSMUSG00000022952
39	11.61	14	23821113	Rai17 CIS2	ENSMUSG00000007817
40	11.46	4	128587790	**Lck**	ENSMUSG00000000409
41	11.27	2	28536129	Gfi1b/**Tsc1**	ENSMUSG00000026815
42	11.13	14	23891838	Rai17 CIS3	ENSMUSG00000007817
43	11.02	7	139458175	**Ccnd1** CIS2	ENSMUSG00000031071
44	10.95	15	83648555	Ttll12	ENSMUSG00000016757
45	10.95	7	67497870	Chd2	ENSMUSG00000066411
46	10.85	4	133073810	Ubxd5 (Socius)	ENSMUSG00000012126
47	10.85	14	109596175	mmu-mir-17	ENSMUSG00000065508
48	10.57	1	165629250	Rcsd1	ENSMUSG00000040723
49	10.53	4	46494177	Coro2a	ENSMUSG00000028337
50	10.36	X	6188924	Eras/**Gata1**	ENSMUSG00000031160
51	10.31	16	92020693	**Runx1** CIS3	ENSMUSG00000022952
52	10	4	135058760	E2F2	ENSMUSG00000018983
53	9.75	6	129919944	Clec12a	ENSMUSG00000053063
54	9.75	2	116859248	Rasgrp1 CIS1	ENSMUSG00000027347
55	9.73	17	33028799	Nfkbil1	ENSMUSG00000042419

56	9.39	4	134975030	Rpl11/Tceb3 CIS2	ENSMUSG00000059291
57	9.21	14	73629775	Elf1 CIS1	ENSMUSG00000036461
58	9.19	11	98306017	Aiolos (Zfpn1a3)/**Erbb2**	ENSMUSG00000018168
59	9.01	15	74955218	Ly6e	ENSMUSG00000022587
60	8.96	2	31991828	Ppapdc3 (D830019K17Rik)**/ Nup214 (BC039282)**	ENSMUSG00000051373
61	8.95	16	92091729	**Runx1** CIS4	ENSMUSG00000022952
62	8.45	11	52072174	Tcf7	ENSMUSG00000000782
63	8.34	8	126125843	Irf2bp2 CIS2	ENSMUSG00000051495
64	8.28	15	62112665	**Myc** CIS3	ENSMUSG00000022346
65	8.13	9	44501321	Bcl9l/Blr1/**Ddx**	ENSMUSG00000063382
66	8.07	7	119678160	**Il21r**/Nsmce1	ENSMUSG00000030745
67	8	12	102594061	CIS without clear target gene nr.2	
68	8	15	73551396	Dennd3	ENSMUSG00000036661
69	7.98	3	29415230	**Evi1**	ENSMUSG00000027684
70	7.98	7	139354527	**Ccnd1** CIS1	ENSMUSG00000031071
71	7.92	5	135638018	Mylc2pl	ENSMUSG00000005474
72	7.9	3	129871915	Lef1/XP_619962.1	ENSMUSG00000027985
73	7.89	1	163990349	Sell/Selp	ENSMUSG00000026581
74	7.87	16	94857193	**Erg**	ENSMUSG00000040732
75	7.85	4	59382155	XP_485387.1 (SUSD1)	ENSMUSG00000038578
76	7.79	11	79250691	**Nf1**	ENSMUSG00000020716
77	7.78	16	31329934	**Tfrc**/Tnk2	ENSMUSG00000022797
78	7.71	10	80817942	Mknk2	ENSMUSG00000020190
79	7.7	13	61798138	Ccrk/Ctsl	ENSMUSG00000021483
80	7.66	17	53932545	Uhrf1	ENSMUSG00000001228
81	7.58	15	62474256	**Myc**/Pvt1 CIS3	ENSMUSG00000022346
82	7.51	11	115245697	Armc7/Atp5h	ENSMUSG00000057219
83	7.49	11	100227067	Fkbp10/Jup	ENSMUSG00000001555
84	7.44	4	62491040	AI597013 (Akna)	ENSMUSG00000039158
85	7.44	4	139828430	Padi1/**Sdhb**	ENSMUSG00000025329
86	7.41	14	116575615	Ebi2/Phghdl1 CIS2	ENSMUSG00000051212
87	7.2	5	147872034	1810059H22Rik/Katnal1 CIS1	ENSMUSG00000066552
88	7.2	11	100664287	Stat5a/Stat5b	ENSMUSG00000004043
89	7.17	6	121361605	Bid CIS2	ENSMUSG00000004446
90	7.14	18	61104445	Tcof1 CIS2	ENSMUSG00000024613
91	7.12	8	83465243	mmu-mir-24-2	ENSMUSG00000065541
92	7.12	11	117147727	**Sept9**	ENSMUSG00000059248
93	7.1	5	104706233	Lrrc5	ENSMUSG00000046079
94	7.05	5	106722658	Gfi1 CIS1	ENSMUSG00000029275
95	7.01	11	116181245	Rnf157 CIS1	ENSMUSG00000020786
96	6.99	4	3881319	**Plag1**	ENSMUSG00000003282
97	6.99	1	191256030	Ints7 (5930412E23Rik)/Dtl	ENSMUSG00000037461
98	6.99	11	98767717	**Rara**	ENSMUSG00000037992
99	6.97	11	82818304	Slfn1/**Nle1**	
100	6.97	6	127826335	**Ccnd2** CIS1	ENSMUSG00000000184
101	6.97	2	30550628	Prrx2/Cstad CIS1	ENSMUSG00000047363
102	6.96	12	108157081	Akt1	ENSMUSG00000001729
103	6.93	10	62471742	Prg1	ENSMUSG00000020077
104	6.89	5	74670796	**Kit**	ENSMUSG00000005672
105	6.88	11	115724245	Recql5	ENSMUSG00000020752
106	6.81	2	26504319	**Notch1**/Egfl7	ENSMUSG00000026923
107	6.8	17	81982560	Haao/Plekhh2	ENSMUSG00000000673
108	6.68	13	37413988	Rreb1 (NP_001013410.2)	ENSMUSG00000039087
109	6.67	2	166826277	Slc9a8	ENSMUSG00000039463
110	6.66	11	54874897	D11Ertd461e (Ccdc69)	ENSMUSG00000049588
111	6.63	3	95161435	Mcl1	ENSMUSG00000038612
112	6.62	6	127924961	**Ccnd2** CIS2	ENSMUSG00000000184
113	6.6	3	93866515	Rorc	ENSMUSG00000028150
114	6.58	5	122293363	Rhof	ENSMUSG00000029449
115	6.48	17	32903185	Nfkbil1/Lta	ENSMUSG00000042419
116	6.46	4	134910289	Rpl11/Tceb3 CIS1	ENSMUSG00000059291
117	6.37	17	26316781	NP_080847.1	ENSMUSG00000062252
118	6.31	5	138376649	3110082I17Rik (Gpr146)/mmu-mir-339	ENSMUSG00000053553
119	6.09	2	32575298	Eng	ENSMUSG00000026814
120	6.05	2	165340868	Prkcbp1/**Sulf2** CIS1	ENSMUSG00000039671
121	6.04	4	132051638	Wasf1/D030015G18Rik/Fgr	ENSMUSG00000028868
122	6.04	16	48648373	Cd47	ENSMUSG00000055447
123	6.04	7	74630640	Iqgap1	ENSMUSG00000030536
124	6	8	83005703	Cd97	ENSMUSG00000002885
125	6	2	13999812	Stam	ENSMUSG00000026718

126	5.99	14	69523068	**Lcp1**	ENSMUSG00000021998
127	5.99	12	103398529	**Bcl11b**	ENSMUSG00000048251
128	5.99	14	23671686	Rai17 CIS1	ENSMUSG00000007817
129	5.99	11	49001295	Mgat1	ENSMUSG00000020346
130	5.98	17	33636505	Ppp1r10/Mrps18b	ENSMUSG00000039220
131	5.98	11	59354627	AA536749	ENSMUSG00000005417
132	5.97	7	121330587	Itgal	ENSMUSG00000030830
133	5.97	9	57756310	Csk/**Cyp1a1**	ENSMUSG00000032312
134	5.94	2	156341571	Sla2	ENSMUSG00000027636
135	5.93	7	135394362	Ifitm2	ENSMUSG00000060591
136	5.93	8	83987843	Lyl1/Nfix	ENSMUSG00000034041
137	5.92	3	85851115	Sh3d19/**Lrba**	ENSMUSG00000028082
138	5.92	3	88368625	Arhgef2	ENSMUSG00000028059
139	5.9	15	80619665	Grap2	ENSMUSG00000042351
140	5.89	1	180313470	Itpkb	ENSMUSG00000038855
141	5.89	3	114450205	Edg1/Q8BYT7_MOUSE	ENSMUSG00000045092
142	5.84	11	98564287	Thrap4	ENSMUSG00000017210
143	5.83	3	87888318	Mef2d	ENSMUSG00000001419
144	5.81	6	125559038	MLF2_MOUSE/Cd4	ENSMUSG00000030120
145	5.81	1	91009086	Ramp1	ENSMUSG00000034353
146	5.81	10	60123944	Psap	ENSMUSG00000004207
147	5.8	16	90609343	Ifnar1	ENSMUSG00000022967
148	5.8	15	82077760	Xrcc6	ENSMUSG00000022471
149	5.79	7	121235400	Spn	ENSMUSG00000051457
150	5.77	11	22704217	Cct4	ENSMUSG00000007739
151	5.77	7	129966482	Ptpre	ENSMUSG00000041836
152	5.76	3	106882405	A930002I21Rik	ENSMUSG00000050179
153	5.76	6	135683425	Gpr19/Cdkn1b	ENSMUSG00000032641
154	5.74	7	62161980	C330024D12Rik	ENSMUSG00000030553
155	5.74	19	4087109	Adbrk1 CIS2	ENSMUSG00000024858
156	5.68	9	110951206	Tmie/Lrrc2/**Als2cl**	ENSMUSG00000049555
157	5.66	7	108154859	Rras2 CIS2	ENSMUSG00000055723
158	5.65	5	63417196	Klf3	ENSMUSG00000029178
159	5.65	2	30585878	Prrx2/Cstad CIS2	ENSMUSG00000047363
160	5.63	8	105403371	Nfatc3	ENSMUSG00000031902
161	5.62	1	133918500	Btg2	ENSMUSG00000020423
162	5.61	1	90880206	Rab17/**Lrrfip1**	ENSMUSG00000026304
163	5.6	15	62361305	**Myc**/Pvt1 CIS2	ENSMUSG00000022346
164	5.57	17	31905576	Tap2	ENSMUSG00000024339
165	5.56	16	54846643	Lrriq2	ENSMUSG00000022604
166	5.52	10	120264582	4921513I03Rik	ENSMUSG00000044544
167	5.5	17	81791745	Mta3/Thada/Haao	ENSMUSG00000055817
168	5.5	18	75510205	Smad7/Dym/**Gm672**	ENSMUSG00000025880
169	5.5	7	40923000	Hps5/Tsg101	ENSMUSG00000014418
170	5.5	10	81060296	Gadd45b	ENSMUSG00000015312
171	5.48	6	121002865	Bid CIS1	ENSMUSG00000004446
172	5.47	14	113351200	Cldn10	ENSMUSG00000022132
173	5.47	13	54774944	H2afy	ENSMUSG00000015937
174	5.47	19	43928459	Wnt8b	ENSMUSG00000036961
175	5.41	1	37777334	Mgat4a	ENSMUSG00000026110
176	5.4	4	117416154	**Mpl**	ENSMUSG00000006389
177	5.4	4	132626060	Pigv/Arid1a CIS1	ENSMUSG00000043257
178	5.4	7	24196140	Nfkbib	ENSMUSG00000030595
179	5.38	15	73760005	Ptp4a3	ENSMUSG00000059895
180	5.38	11	86313637	Rps6kb1/mmu-mir-21	ENSMUSG00000020516
181	5.33	7	33352770	1600014C10Rik	ENSMUSG00000054676
182	5.31	16	28770673	Hes1	ENSMUSG00000022528
183	5.3	15	63669425	**Myc**/Pvt1 CIS5	ENSMUSG00000022346
184	5.29	10	79977684	Ptbp1/**Fstl3**	ENSMUSG00000006498
185	5.29	2	165410485	Prkcbp1/**Sulf2** CIS2	ENSMUSG00000039671
186	5.23	1	86294310	Ptma CIS2	ENSMUSG00000026238
187	5.23	5	136645306	Hrbl	ENSMUSG00000029722
188	5.19	X	135308787	Irs4	ENSMUSG00000054667
189	5.19	15	63279275	**Myc**/Pvt1 CIS4	ENSMUSG00000022346
190	5.18	7	123850650	Fgfr2	ENSMUSG00000030849
191	5.18	2	152235398	Bcl2l1	ENSMUSG00000007659
192	5.16	19	41386799	Arhgap19/Frat2	ENSMUSG00000025154
193	5.13	13	108811918	Il6st	ENSMUSG00000021756
194	5.12	7	74001090	Sema4b	ENSMUSG00000030539
195	5.1	9	72522851	ENSMUSESTG00000012933	ENSMUSESTG00000012

					933
196	5.09	7	119537370	Nsmce1	ENSMUSG00000030750
197	5.09	4	128793830	Khdrbs1/ptp4a2	ENSMUSG00000028790
198	5.05	6	147440275	Fgfr1op2/Itpr5	ENSMUSG00000040242
199	5.02	8	110893158	Znrf1	ENSMUSG00000033545
200	5.01	5	123654737	6330548G22Rik/**Sbno1**	ENSMUSG00000029402
201	5	17	25350322	**Hmga1**	ENSMUSG00000046711
202	5	1	138025291	Ptprc	ENSMUSG00000026395
203	5	5	148367308	4930588N13Rik	ENSMUSG00000029660
204	5	5	32170510	**Fgfr3**	ENSMUSG00000054252
205	4.99	8	125950774	Irf2bp2 CIS1	ENSMUSG00000051495
206	4.99	12	110690521	ENSMUSG00000066294	ENSMUSG00000066294
207	4.98	5	135184914	Tmem142b (A730041O15Rik)	ENSMUSG00000039747
208	4.98	19	6187971	Rasgrp2/**Men1**	ENSMUSG00000032946
209	4.97	2	11551388	Il2ra	ENSMUSG00000026770
210	4.97	2	44857938	Zfhx1b CIS1	ENSMUSG00000026872
211	4.97	1	133102528	Plekha6	ENSMUSG00000041757
212	4.97	X	98219402	Cnbp2	ENSMUSG00000031330
213	4.96	6	99853435	Foxp1	ENSMUSG00000030067
214	4.94	11	86134747	Thrap1	ENSMUSG00000034297
215	4.94	9	44227181	**Cbl**	ENSMUSG00000034342
216	4.94	6	72720046	Vamp5	ENSMUSG00000055545
217	4.94	4	131937555	Fgr	ENSMUSG00000028874
218	4.93	5	110555222	C130026L21Rik	ENSMUSG00000052848
219	4.93	5	99658936	Plac8 CIS2	ENSMUSG00000029322
220	4.92	5	99594376	Plac8 CIS1	ENSMUSG00000029322
221	4.92	19	28737299	C030046E11Rik (KIAA1432)	ENSMUSG00000038658
222	4.91	6	91554672	Nup210	ENSMUSG00000030091
223	4.91	9	123788343	Ccr9/Lztfl1	ENSMUSG00000029530
224	4.91	2	91340558	Arhgap1	ENSMUSG00000027247
225	4.9	4	154563686	Ttll10 (4833412E22Rik)/mmu-mir-200b	ENSMUSG00000029074
226	4.9	5	112944815	Selpl	ENSMUSG00000048163
227	4.9	2	167313066	Ptpn1/AI840826	ENSMUSG00000027540
228	4.9	17	13683105	Chd1	ENSMUSG00000023852
229	4.88	11	106496557	Pecam1 CIS1	ENSMUSG00000020717
230	4.88	10	81462052	Tbxa2r	ENSMUSG00000034881
231	4.85	18	70797625	Mbd2	ENSMUSG00000024513
232	4.81	13	50518384	Sema4d CIS1	ENSMUSG00000021451
233	4.81	17	42284355	Runx2	ENSMUSG00000039153
234	4.8	19	4012079	Adrbk1 CIS1	ENSMUSG00000024858
235	4.79	2	6624010	Cugbp2	ENSMUSG00000002107
236	4.76	2	126844028	Dusp2	ENSMUSG00000027368
237	4.76	11	86624557	Dhx40/**Cltc**	ENSMUSG00000018425
238	4.74	6	128010523	**Ccnd2** CIS3	ENSMUSG00000000184
239	4.74	7	134602200	E430002D04Rik	ENSMUSG00000025461
240	4.74	11	119658667	Raptor	ENSMUSG00000025583
241	4.72	5	138780286	Mafk	ENSMUSG00000018143
242	4.71	10	43934712	Rtn4ip1	ENSMUSG00000019864
243	4.7	4	8846030	CIS without clear target gene nr.1	
244	4.7	14	73877874	Elf1 CIS2	ENSMUSG00000036461
245	4.65	4	105872210	Ssbp3/Thea CIS2	ENSMUSG00000061887
246	4.64	9	114405251	Glb1	ENSMUSG00000045594
247	4.64	17	33739737	Prr3	ENSMUSG00000038500
248	4.64	16	4228333	Tcfap4	ENSMUSG00000005718
249	4.63	1	86244930	Ptma CIS1	ENSMUSG00000026238
250	4.61	15	95822465	Tmem16f/Dbx2	ENSMUSG00000064210
251	4.61	3	95453515	Otud7b (Za20d1) CIS2	ENSMUSG00000038495
252	4.59	4	105792860	Ssbp3/Thea CIS1	ENSMUSG00000061887
253	4.58	1	171604037	Cd48	ENSMUSG00000015355
254	4.57	10	93098682	Pctk2	ENSMUSG00000020015
255	4.55	5	147922096	1810059H22Rik/Katnal1 CIS2	ENSMUSG00000066552
256	4.53	1	171853080	Slamf6	ENSMUSG00000015314
257	4.52	6	125385235	mmu-mir-200c&141	ENSMUSG00000065462
258	4.5	3	103209145	Hipk1	ENSMUSG00000008730
259	4.5	6	125889505	Tnfrsf7/Tapbp1	ENSMUSG00000030336
260	4.48	11	106576087	Pecam1 CIS2	ENSMUSG00000020717
261	4.48	14	39096025	Tspan14	ENSMUSG00000037824
262	4.47	18	60982195	Tcof1 CIS1	ENSMUSG00000024613
263	4.46	12	83704299	2310044G17Rik	ENSMUSG00000034157
264	4.44	5	116275696	TAOK3 (A430105I05Rik)/Suds3 (2400003N08Rik)	ENSMUSG00000061288

265	4.43	3	151933285	St6galnac5	ENSMUSG00000039037
266	4.42	16	92989123	Morc3	ENSMUSG00000039456
267	4.38	9	44995584	Cd3e/Cd3d/**Mll**	ENSMUSG00000032093
268	4.38	18	65143165	Nedd4l	ENSMUSG00000024589
269	4.38	13	50636308	Sema4d CIS2	ENSMUSG00000021451
270	4.37	11	100707907	Stat3/Sta5a/Stat5b	ENSMUSG00000004040
271	4.37	18	39252235	**Arhgap26**	ENSMUSG00000036452
272	4.33	5	64712686	**Rhoh** CIS1	ENSMUSG00000029204
273	4.33	11	68741802	Aurkb/**Per1**	ENSMUSG00000020897
274	4.32	15	81568955	**Ep300**	ENSMUSG00000055024
275	4.31	1	136101000	Kif21b/5730559C18Rik	ENSMUSG00000041642
276	4.29	6	52788955	Tax1bp1/**Jazf1**(AI591476)	ENSMUSG00000004535
277	4.28	18	85053295	Fbxo15	ENSMUSG00000034391
278	4.27	2	62255318	Dpp4	ENSMUSG00000035000
279	4.26	12	108902265	Igh-6 CIS1	ENSMUSG00000054328
280	4.25	15	103321042	Nfe2	ENSMUSG00000058794
281	4.23	8	94263863	Gpr56	ENSMUSG00000031785
282	4.23	4	43375580	Cd72	ENSMUSG00000028459
283	4.23	4	134782670	Cnr2	ENSMUSG00000062585
284	4.23	14	67474050	Chc1l	ENSMUSG00000022106
285	4.22	8	122782319	Mc1r	ENSMUSG00000044070
286	4.22	1	178285800	Hnrpu	ENSMUSG00000039630
287	4.16	10	93313812	Elk3	ENSMUSG00000008398
288	4.15	11	116237707	Rnf157 CIS2	ENSMUSG00000020786
289	4.15	5	136690778	Hrbl/6430598A04Rik	ENSMUSG00000029722
290	4.13	9	109100351	Scotin	ENSMUSG00000025647
291	4.12	6	31166635	mmu-mir-29a	ENSMUSG00000065610
292	4.11	10	19516030	Il22ra2	ENSMUSG00000039760
293	4.1	7	74372280	Crtc3 (2610312F20Rik)	ENSMUSG00000030527
294	4.1	14	116536015	Ebi2/Phghdl1 CIS1	ENSMUSG00000051212
295	4.08	9	108044711	Ube1l	ENSMUSG00000032596
296	4.07	15	36452345	Rnf19	ENSMUSG00000022280
297	4.06	11	114982131	Rab37	ENSMUSG00000020732
298	4.06	3	95416724	Otud7b (Za20d1) CIS1	ENSMUSG00000038495
299	4.06	7	74247390	Fes	ENSMUSG00000053158
300	4.05	2	103641698	**Lmo2**	ENSMUSG00000032698
301	4.05	19	3940889	Ptprcap	ENSMUSG00000045826
302	4.05	3	9883135	Pag1	ENSMUSG00000027508
303	4.04	7	16348560	Mark4/4933417E01Rik	ENSMUSG00000030397
304	4.04	4	132781610	Pigv/Arid1a CIS3	ENSMUSG00000043257
305	4.03	X	11481263	Ddx3x	ENSMUSG00000000787
306	4.03	2	163105208	Tde1/Pkig	ENSMUSG00000017707
307	4.03	X	18857340	Rbm10	ENSMUSG00000031060
308	4.03	8	122803223	Q60588_MOUSE (Gag)	ENSMUSG00000057475
309	4.02	18	4338175	Map3k8 (Tpl-2)	ENSMUSG00000024235
310	4.01	7	120756180	Coro1a	ENSMUSG00000030707
311	4	16	16742627	Vpreb2	ENSMUSG00000059280
312	4	17	49415925	Satb1	ENSMUSG00000023927
313	4	12	106173728	**Hspca**	ENSMUSG00000021270
314	4	2	126434497	SPPL2a (2010106G01Rik)	ENSMUSG00000027366
315	4	12	109703608	Igh-6 CIS2	ENSMUSG00000054328
316	4	2	45043418	Zfhx1b CIS2	ENSMUSG00000026872
317	4	11	77244457	Git1	ENSMUSG00000011877
318	4	8	10297145	3930402G23Rik CIS1	ENSMUSG00000038917
319	3.99	11	107492756	Helz	ENSMUSG00000020721
320	3.99	11	79340398	Rab11fip4	ENSMUSG00000017639
321	3.99	13	97003691	Ccnb1/Slc30a5	ENSMUSG00000041431
322	3.99	10	42999792	Scml4	ENSMUSG00000044770
323	3.99	18	50245334	Tnfaip8	ENSMUSG00000062210
324	3.99	7	73843054	5430400N05Rik	ENSMUSG00000048897
325	3.99	19	31587959	Tmem23	ENSMUSG00000040451
326	3.99	11	67927204	Pik3r5/Ntn1 CIS1	ENSMUSG00000020901
327	3.99	11	57827225	Cnot8	ENSMUSG00000020515
328	3.99	17	44280165	NP_035325.1	ENSMUSG00000036858
329	3.99	4	132717374	Pigv/Arid1a CIS2	ENSMUSG00000043257
330	3.99	4	134014400	Tmem50a	ENSMUSG00000028822
331	3.99	7	95200245	Fcshd2	ENSMUSG00000030691
332	3.99	9	7246601	Mmp13	ENSMUSG00000050578
333	3.99	2	27054638	C630035N08Rik	ENSMUSG00000009216
334	3.98	17	12963705	Phf10/Tcte3	ENSMUSG00000023883

335	3.98	17	45563625	1700122O11Rik/Foxp4	ENSMUSG00000042494
336	3.98	13	27994348	ENSMUSG00000069257	ENSMUSG00000069257
337	3.98	3	102814255	Ptpn22/**Trim33**	ENSMUSG00000027843
338	3.98	11	48834004	Tgtp/Ifi47	ENSMUSG00000040335
339	3.98	3	100704312	Cd2	ENSMUSG00000027863
340	3.98	6	8460029	Glcci1	ENSMUSG00000029638
341	3.98	15	78646266	Rac2/**Tmprss6**	ENSMUSG00000033220
342	3.98	7	95441460	Stard10	ENSMUSG00000030688
343	3.97	18	35181355	Hspa9a	ENSMUSG00000024359
344	3.97	15	102462245	Sp1	ENSMUSG00000001280
345	3.97	17	32751830	Clic1_MOUSE	ENSMUSG00000007041
346	3.97	10	20926812	Myb/Ahi-1 CIS1	ENSMUSG00000019982

Table S3. Enrichment for mutation of canonical pathways

Canonical pathway	p-value
Predicted target gene of CISs	
T Cell Receptor Signaling	5,37E-09
GM-CSF Signaling	2,34E-08
ERK/MAPK Signaling	4,79E-08
B Cell Receptor Signaling	5,50E-08
PTEN Signaling	4,90E-06
PI3K/AKT Signaling	7,08E-06
IL-2 Signaling	1,38E-05
Insulin Receptor Signaling	2,00E-05
JAK/Stat Signaling	2,88E-05
IL-4 Signaling	4,47E-05
Integrin Signaling	7,59E-05
Leukocyte Extravasation Signaling	1,82E-04
SAPK/JNK Signaling	5,62E-04
NF-κB Signaling	1,32E-03
Cell Cycle: G1/S Checkpoint Regulation	1,66E-03
Nearest gene to CISs	
T Cell Receptor Signaling	3,55E-05
Cell Cycle: G1/S Checkpoint Regulation	6,03E-05
GM-CSF Signaling	8,91E-05
ERK/MAPK Signaling	1,32E-04
JAK/Stat Signaling	7,08E-04
B Cell Receptor Signaling	2,14E-03
IL-2 Signaling	3,72E-03
Neuregulin Signaling	4,07E-03
Interferon Signaling	4,27E-03
PPAR Signaling	4,27E-03
Nitric Oxide Signaling in the Cardiovascular System	5,62E-03
IL-4 Signaling	6,92E-03
Notch Signaling	1,02E-02
EGF Signaling	2,00E-02
p38 MAPK Signaling	2,63E-02
Cancer Gene Census genes	
PI3K/AKT Signaling	6,30957E-18
Apoptosis Signaling	6,30957E-11
NF-κB Signaling	7,94328E-11
TGF-β Signaling	1E-10
PTEN Signaling	3,71535E-10
SAPK/JNK Signaling	4,67735E-10
Cell Cycle: G1/S Checkpoint Regulation	5,49541E-09
PPAR Signaling	1,47911E-08
Ephrin Receptor Signaling	2,88403E-08
ERK/MAPK Signaling	3,0903E-08
B Cell Receptor Signaling	6,16595E-08
Integrin Signaling	7,94328E-08
T Cell Receptor Signaling	1,1749E-07
PDGF Signaling	1,47911E-07
Insulin Receptor Signaling	2,04174E-07

Table S3 Enrichment for mutation of canonical pathways. Enrichment of CIS candidate target genes for genes mapping to canonical pathways was determined using the ‘canonical pathway’ option of the Ingenuity Pathway Analysis software. As a comparison, the nearest gene to each of the 346 CISs was selected as well as the complete set of murine orthologs of Cancer Gene Census genes and both sets were examined for enrichment of genes mapping to canonical pathways.

Table S4. Overlap of individual panel CISs between genotypes

25 $p19^{-/-}$+$p53^{-/-}$+wt	22 $p19^{-/-}$+wt	11 $p19^{-/-}$+$p53^{-/-}$	2 $p53^{-/-}$+wt	55 $p19^{-/-}$	38 wt	47 $p53^{-/-}$
Gfi1 CIS2	Ikaros (Zfpn1a1)	Zhfx1a	Rcsd1	Notch1 CIS2	**Myb/Ahi-1 CIS3**	Ints7 (5930412E23Rik)/Dtl
Myc CIS1	Rai17 CIS3	Runx1 CIS1	Erg	Eras/Gata1	ETS1/Fli1	Znrf1
Rasgrp1 CIS2	Rai17 CIS2	FLT3		Smg6/rno-mir-132&212/Ovca2/HIC1 CIS2	Evi1	Mylc2pl
Rras2 CIS1	E2F2	mmu-mir-142		Coro2a	Uhrf1	Xrcc6
Notch1 CIS1	Evi1/Mds1	Smg6/rno-mir-132&212/Ovca2/HIC1 CIS1		Chd2	Ptma CIS1	Eng
Ccnd3	Ttll12	Runx1 CIS5		Ppapdc3 (D830019K17Rik) /Nup214 (BC039282)	Rai17 CIS1	Thrap1
Myc/Pvt1 CIS1	Nos2/Ksr/Lgals9	Gfi1b/Tsc1		Stat5a/Stat5b	Phf10/Tcte3	Rhof
Pim1 CIS2	mmu-mir-17	Ubxd5		Myc CIS3	Prg1	Ccrk/Ctsl
Myb/Ahi-1 CIS4	Runx1 CIS6	Nfatc3		Fkbp10/Jup	CIS without clear target gene nr.2	Recql5
Pik3r5/Ntn1 CIS2	Rasgrp1 CIS1	Armc7/Atp5h		Akt1	**4921504K03Rik***	Sp1
Jundm2 CIS2	Clec12a	AI597013 (AKNA)		Stam	Arhgap19/Frat2	Chc1l
mmu-mir-106a	Ly6e			Ap2b1	Rreb1 (NP_001013410.2)	Sell/Selp
Myc CIS2	Rpl11/Tceb3 CIS2			AA536749	Bcl9l/Blr1/Ddx	mmu-mir-24-2
Lfng	Ccnd1 CIS2			Tap2	Smad7/Dym/Gm672	Grap2
Myb/Ahi-1 CIS2	Irf2bp2 CIS2			Rras2 CIS2	**Rapgef3**	Btg2
CCR7	Lck			Runx1 CIS4	Haao/Plekhh2	Itgal
Nmyc1	Pim1 CIS1			Runx1 CIS3	Foxp1	Ssbp3/Thea CIS1
Jundm2 CIS1	Il21r/Nsmce1			Tcf7	Nsmce1	1810059H22Rik/Katnal1 CIS1
Pik3cd	Kit			C330024D12Rik	Sept9	Rps6kb1/mmu-mir-21
Cbfa2t3h	Dennd3			Elf1 CIS1	Cd97	Mknk2
Runx1 CIS2	Plag1			Rpl11/Tceb3 CIS1	Myc/Pvt1 CIS4	Nfkbil1/Lta
Elf4	Smg6			Mpl	Gfi1 CIS1	Cd47
Nfkbil1				Lrrc5	Bcl2l1	Bid CIS1
Pim2				Ccnd1 CIS1	Hps5/Tsg101	**ENSMUSESTG00000024159***
Aiolos (Zfpn1a3)/Erbb2				Sema4b	**Rhoh CIS2***	**E2F8***
				Wasf1/D030015G18Rik/Fgr	Ptbp1/Fstl3	Ccnd2 CIS2
				Coro1a	D11Ertd461e	Tcof1 CIS2
				Rnf157 CIS1	Edg1/Q8BYT7_MOUSE	Hrbl/6430598A04Rik
				Tmie/Lrrc2/Als2cl	Irf2bp2 CIS1	Cd48
				ENSMUSG00000066294	Ddx3x	Tmem16f/Dbx2
				Ppp1r10/Mrps18b	Ptprc	XP_485387.1 (SUSD1)
				Gadd45b	**3930402G23Rik CIS2***	**Lrmp***
				NF1	Zfhx1b CIS1	Itpkb
				Sh3d19/Lrba	Nedd4l	**1810045K07Rik**
				Psap	Bcl11b	**NP_808253.1***
				Nup210	Ccnd2 CIS1	**Agps***
				Padi1/Sdhb	Igh-6 CIS1	E430002D04Rik
				Ifitm2	**Il16***	Cd3e/Cd3d
				Myc/Pvt1 CIS3		Raptor
				Cct4		Satb1
				Ccnd2 CIS3		Tmem142b (A730041O15Rik)
				Ebi2/Phghdl1 CIS2		MLF2_MOUSE/Cd4
				**Nxf1***		Rasgrp2/Men1
				Lrriq2		Ptpn22/Trim33
				**Btk***		**Tinagl***
				Otud7b (Za20d1) CIS2		Plekha6
				Fes		**1300010F03Rik***
				TAOK3 (A430105I05Rik)/Suds3 (2400003N08Rik)		
				**D1Bwg0491e***		
				Prrx2/Cstad CIS1		
				Lyl1/Nfix		
				Notch1/Egfl7		
				CIS without clear target gene nr.3		
				Cd72		
				H2afy		

Table S4 Overlap of individual panel CISs between genotypes. Candidate target genes of CISs found in tumors of the indicated genotypes. New CISs that are only found as individual genotype CISs and not when all insertions are combined are in bold. CISs exclusively mutated in one of the three genotypes are indicated by an asterisk (*). CISs are listed in order of decreasing significance: target genes of the most significant CISs are on the top of the list.

Table S5. Enrichment for mutation of canonical pathways in individual panels or combinations of panels.

Pathway	Genotype	p-value	Gene
IL-2 Signaling	$p19^{ARF-/-}$	5.50E-03	STAT5A,AKT1
	wt	NA	
	$p53^{-/-}$	NA	
	wt & $p19^{ARF-/-}$	1.70E-03	STAT5A,LCK,AKT1
	wt & $p53^{-/-}$	NA	
	$p19^{ARF-/-}$ & $p53^{-/-}$	2.63E-02	STAT5A,AKT1
JAK/Stat Signaling	$p19^{ARF-/-}$	6.92E-03	STAT5A,AKT1
	wt	NA	
	$p53^{-/-}$	NA	
	wt & $p19^{ARF-/-}$	2.88E-02	STAT5A,AKT1
	wt & $p53^{-/-}$	NA	
	$p19^{ARF-/-}$ & $p53^{-/-}$	3.16E-02	STAT5A,AKT1
Neuregulin Signaling	$p19^{ARF-/-}$	1.48E-02	STAT5A,AKT1
	wt	NA	
	$p53^{-/-}$	1.66E-01	RPS6KB1
	wt & $p19^{ARF-/-}$	6.03E-02	STAT5A,AKT1
	wt & $p53^{-/-}$	2.77E-01	RPS6KB1
	$p19^{ARF-/-}$ & $p53^{-/-}$	8.51E-03	STAT5A,RPS6KB1,AKT1
p53 Signaling	$p19^{ARF-/-}$	1.48E-02	AKT1,GADD45B
	wt	1.28E-01	BCL2L1
	$p53^{-/-}$	NA	
	wt & $p19^{ARF-/-}$	6.61E-04	BCL2L1,AKT1,GADD45B,CCND1
	wt & $p53^{-/-}$	2.77E-01	BCL2L1
	$p19^{ARF-/-}$ & $p53^{-/-}$	8.51E-03	AKT1,GADD45B,CCND2
Fc Epsilon RI Signaling	$p19^{ARF-/-}$	1.91E-02	BTK,AKT1
	wt	NA	
	$p53^{-/-}$	1.87E-01	GRAP2
	wt & $p19^{ARF-/-}$	7.41E-02	BTK,AKT1
	wt & $p53^{-/-}$	3.09E-01	GRAP2
	$p19^{ARF-/-}$ & $p53^{-/-}$	1.20E-02	BTK,AKT1,GRAP2
Insulin Receptor Signaling	$p19^{ARF-/-}$	2.95E-02	AKT1,PPP1R10
	wt	NA	
	$p53^{-/-}$	2.75E-02	RPS6KB1,RAPTOR
	wt & $p19^{ARF-/-}$	1.11E-01	AKT1,PPP1R10
	wt & $p53^{-/-}$	7.94E-02	RPS6KB1,RAPTOR
	$p19^{ARF-/-}$ & $p53^{-/-}$	3.09E-03	RPS6KB1,AKT1,PPP1R10,RAPTOR
Endoplasmic Reticulum Stress Pathway	$p19^{ARF-/-}$	3.80E-02	TAOK3
	wt	NA	
	$p53^{-/-}$	NA	
	wt & $p19^{ARF-/-}$	7.94E-02	TAOK3
	wt & $p53^{-/-}$	NA	
	$p19^{ARF-/-}$ & $p53^{-/-}$	8.32E-02	TAOK3
B Cell Receptor Signaling	$p19^{ARF-/-}$	3.98E-02	BTK,AKT1
	wt	1.51E-03	ETS1,PTPRC,BCL2L1
	$p53^{-/-}$	2.65E-01	RPS6KB1
	wt & $p19^{ARF-/-}$	5.50E-04	ETS1,BTK,PTPRC,BCL2L1,AKT1
	wt & $p53^{-/-}$	2.14E-03	ETS1,PTPRC,BCL2L1,RPS6KB1
	$p19^{ARF-/-}$ & $p53^{-/-}$	5.50E-03	BTK,RPS6KB1,AKT1,NFATC3
Axonal Guidance Signaling	$p19^{ARF-/-}$	4.90E-02	AKT1,FES,SEMA4B

	wt	NA	
	$p53^{-/-}$	NA	
	wt & $p19^{ARF-/-}$	2.56E-01	AKT1,FES,SEMA4B
	wt & $p53^{-/-}$	NA	
	$p19^{ARF-/-}$ & $p53^{-/-}$	1.15E-01	AKT1,FES,NFATC3,SEMA4B
ERK/MAPK Signaling	$p19^{ARF-/-}$	6.76E-02	PPP1R10,ELF1
	wt	3.80E-02	ETS1,RAPGEF3
	$p53^{-/-}$	3.41E-01	MKNK2
	wt & $p19^{ARF-/-}$	1.29E-02	ETS1,PPP1R10,RAPGEF3,ELF1
	wt & $p53^{-/-}$	3.80E-02	ETS1,RAPGEF3,MKNK2
	$p19^{ARF-/-}$ & $p53^{-/-}$	7.08E-02	PPP1R10,MKNK2,ELF1
GM-CSF Signaling	$p19^{ARF-/-}$	1.21E-01	AKT1
	wt	3.89E-03	ETS1,BCL2L1
	$p53^{-/-}$	NA	
	wt & $p19^{ARF-/-}$	7.59E-06	ETS1,BCL2L1,AKT1,PIM1,CCND1
	wt & $p53^{-/-}$	2.09E-02	ETS1,BCL2L1
	$p19^{ARF-/-}$ & $p53^{-/-}$	3.39E-02	AKT1,RUNX1
IL-4 Signaling	$p19^{ARF-/-}$	1.27E-01	AKT1
	wt	NA	
	$p53^{-/-}$	1.23E-01	RPS6KB1
	wt & $p19^{ARF-/-}$	2.51E-01	AKT1
	wt & $p53^{-/-}$	2.09E-01	RPS6KB1
	$p19^{ARF-/-}$ & $p53^{-/-}$	3.47E-03	RPS6KB1,AKT1,NFATC3
T Cell Receptor Signaling	$p19^{ARF-/-}$	2.00E-01	BTK
	wt	1.49E-01	PTPRC
	$p53^{-/-}$	1.95E-02	CD3E,GRAP2
	wt & $p19^{ARF-/-}$	1.26E-03	BTK,PTPRC,LCK,RASGRP1
	wt & $p53^{-/-}$	6.61E-03	PTPRC,CD3E,GRAP2
	$p19^{ARF-/-}$ & $p53^{-/-}$	1.55E-03	BTK,CD3E,NFATC3,GRAP2
PTEN Signaling	$p19^{ARF-/-}$	2.05E-01	AKT1
	wt	1.53E-01	BCL2L1
	$p53^{-/-}$	1.99E-01	RPS6KB1
	wt & $p19^{ARF-/-}$	1.26E-02	BCL2L1,AKT1,CCND1
	wt & $p53^{-/-}$	5.89E-02	BCL2L1,RPS6KB1
	$p19^{ARF-/-}$ & $p53^{-/-}$	9.33E-02	RPS6KB1,AKT1
PI3K/AKT Signaling	$p19^{ARF-/-}$	2.59E-01	AKT1
	wt	1.96E-01	BCL2L1
	$p53^{-/-}$	2.53E-01	RPS6KB1
	wt & $p19^{ARF-/-}$	2.57E-02	BCL2L1,AKT1,CCND1
	wt & $p53^{-/-}$	9.33E-02	BCL2L1,RPS6KB1
	$p19^{ARF-/-}$ & $p53^{-/-}$	1.45E-01	RPS6KB1,AKT1
Leukocyte Extravasation Signaling	wt	3.31E-02	RAPGEF3,RHOH
	$p53^{-/-}$	3.21E-01	ITGAL
	wt & $p19^{ARF-/-}$	5.25E-02	BTK,RAPGEF3,RHOH
	wt & $p53^{-/-}$	3.16E-02	RAPGEF3,ITGAL,RHOH
	$p19^{ARF-/-}$ & $p53^{-/-}$	2.24E-01	BTK,ITGAL
Cell Cycle: G1/S Checkpoint Regulation	$p19^{ARF-/-}$	NA	
	wt	NA	
	$p53^{-/-}$	NA	
	wt & $p19^{ARF-/-}$	2.51E-02	CCND1,E2F2
	wt & $p53^{-/-}$	NA	
	$p19^{ARF-/-}$ & $p53^{-/-}$	2.30E-01	CCND2

Table S5. Enrichment for mutation of canonical pathways in individual panels or combinations of panels was determined using the 'canonical pathway' option of the Ingenuity Pathway Analysis software. CIS candidate target genes as listed in Table S4 for the respective (combinations of) genotypes are used for this analysis. In case two candidate genes are assigned to a CIS, the first gene is used as this is considered to be the most likely target gene. Genotypes not having any CIS mapping to a particular pathway are labeled 'NA'.
of decreasing significance: target genes of the most significant CISs are on the top of the list.

Tables S6, S7, and S8, please see separate Excel files.

Table S6 Co-occurrence and mutual exclusivity of 300 kb CISs. Co-occurrence or mutual exclusivity of insertions in 300 kb CISs in tumors was determined using contingency tables. For each CIS pair the interaction was tested with either CIS A or CIS B as the ‘predisposing’ and the other CIS as the ‘subsequent’ event. All CIS pairs yielding one or two p-values below 0.05 are listed and ranked on the basis of lowest p-value. Positive p-values indicate co-occurrence. Negative p-values indicate mutual exclusivity.

Table S7. Co-occurrence and mutual exclusivity of 5 kb CISs. Co-occurrence or mutual exclusivity of insertions in 5 kb CISs in tumors was determined using contingency tables. For each CIS pair the interaction was tested with either CIS A or CIS B as the ‘predisposing’ and the other CIS as the ‘subsequent’ event. All CIS pairs yielding one or two p-values below 0.05 are listed and ranked on the basis of lowest p-value. Positive p-values indicate co-occurrence. Negative p-values indicate mutual exclusivity.

Table S8. Co-occurrence and mutual exclusivity of 30 kb CISs. Co-occurrence or mutual exclusivity of insertions in 30 kb CISs in tumors was determined using contingency tables. For each CIS pair the interaction was tested with either CIS A or CIS B as the ‘predisposing’ and the other CIS as the ‘subsequent’ event. All CIS pairs yielding one or two p-values below 0.05 are listed and ranked on the basis of lowest p-value. Positive p-values indicate co-occurrence. Negative p-values indicate mutual exclusivity.

Table S9. Genes with 3 or more insertions within gene

Chr.	Position	Genename	Insertions within gene
17	45023592	ENSMUSG00000034165 - Ccnd3	148
16	91766299	ENSMUSG00000022952 - Runx1	51
11	11580718	ENSMUSG00000018654 - Zfpn1a1 (Ikaros)	50
5	1,07E+08	ENSMUSG00000011831 - Evi5	50
2	26390199	ENSMUSG00000026923 - Notch1	47
11	74651520	ENSMUSG00000038290 – Smg6 (Est1a)	35
12	12294435	ENSMUSG00000037169 - Mycn	35
10	20889304	ENSMUSG00000019986 - Ahi1	31
12	82468669	ENSMUSG00000034271 - Jundm2	30
X	6098173	ENSMUSG00000050227 -	27
3	29353351	ENSMUSG00000027684 - Evi1	24
17	27300659	ENSMUSG00000024014 - Pim1	22
5	1,46E+08	ENSMUSG00000042817 - Flt3	21
5	1,07E+08	ENSMUSG00000029275 - Gfi1	19
14	23834466	ENSMUSG00000007817 - Rai17	17
6	1,25E+08	ENSMUSG00000030122 -	17
4	1,35E+08	ENSMUSG00000059291 -	17
15	61997535	ENSMUSG00000022346 - Myc	14
14	1,16E+08	ENSMUSG00000041765 - Phgdhl1	14
4	46453064	ENSMUSG00000028337 - Coro2a	13
11	98965287	ENSMUSG00000037944 - Ccr7	13
X	6081058	ENSMUSG00000031154 - Otud5	12
10	81429154	ENSMUSG00000034902 - Pip5k1c	11
11	1,15E+08	ENSMUSG00000045775 -	11
11	98288074	ENSMUSG00000018168 - Zfpn1a3 (Aiolos)	11
17	4X01401	ENSMUSG00000039153 -	11
16	94768855	ENSMUSG00000040732 - Erg	11
4	1,33E+08	ENSMUSG00000012126 - Ubxd5	10
4	1,35E+08	ENSMUSG00000018983 - E2f2	10
4	1,29E+08	ENSMUSG00000000409 - Lck	10
11	1,06E+08	ENSMUSG000000X717 - Pecam1	10
11	68157780	ENSMUSG000000X901 - Pik3r5	9
4	96557051	ENSMUSG00000028565 - Nfia	9
18	39216930	ENSMUSG00000036452 - Arhgap26	9
11	1,17E+08	ENSMUSG00000059248 - Sept9	9
5	1,38E+08	ENSMUSG00000053553 - 3110082I17Rik	9
8	69668767	ENSMUSG00000055553 - 2810422J05Rik	9
14	23817968	ENSMUSG00000068668 -	9
11	79065552	ENSMUSG000000X716 - Nf1	8
15	73541042	ENSMUSG00000036661 - Dennd3	8
1	1,8E+08	ENSMUSG00000038855 - Itpkb	8
17	26287388	ENSMUSG00000062252 -	8
2	1,52E+08	ENSMUSG00000007659 - Bcl2l1	8
2	1,65E+08	ENSMUSG00000039671 - Prkcbp1	8
11	79239045	ENSMUSG00000046628 - Evi2b	8
11	48657496	ENSMUSG00000048852 - Q5NCB2_MOUSE	8
7	6X90250	ENSMUSG00000066416 -	8
8	1,14E+08	ENSMUSG00000004637 - Wwox	7
14	25253105	ENSMUSG00000021895 -	7
3	93860610	ENSMUSG00000028150 - Rorc	7
4	1,4E+08	ENSMUSG00000028927 - Padi2	7
8	1,11E+08	ENSMUSG00000033545 - Znrf1	7
5	1,07E+08	ENSMUSG00000033773 - AW060X7	7
18	75253962	ENSMUSG00000035765 - Dym	7
11	98758786	ENSMUSG00000037992 - Rara	7
17	42281337	ENSMUSG00000038954 - Supt3h	7
11	48731727	ENSMUSG00000040328 - Olfr56	7
7	1,3E+08	ENSMUSG00000041836 - Ptpre	7
15	80580364	ENSMUSG00000068166 -	7

10	62393645	ENSMUSG00000069613 -	7
11	1,01E+08	ENSMUSG00000004040 - Stat3	7
11	98357626	ENSMUSG00000017210 - Thrap4	7
11	1,16E+08	ENSMUSG000000X752 - Recql5	7
1	37738795	ENSMUSG00000026110 - Mgat4a	7
2	1,17E+08	ENSMUSG00000027347 - Rasgrp1	7
14	39051256	ENSMUSG00000037824 - Tspan14	7
8	69656628	ENSMUSG00000070003 - Ssbp4	7
11	1,15E+08	ENSMUSG00000016940 -	6
11	48997088	ENSMUSG000000X346 - Mgat1	6
14	69480275	ENSMUSG00000021998 - Lcp1	6
15	8X66237	ENSMUSG00000022471 - Xrcc6	6
11	1,19E+08	ENSMUSG00000025583 - 4932417H02Rik	6
1	90818347	ENSMUSG00000026305 -	6
1	1,64E+08	ENSMUSG00000026581 - Sell	6
3	88365124	ENSMUSG00000028059 - Arhgef2	6
3	85829277	ENSMUSG00000028082 - Sh3d19	6
6	41199334	ENSMUSG00000029881 - 5830405F06Rik	6
8	1,05E+08	ENSMUSG00000031902 - Nfatc3	6
1	91003063	ENSMUSG00000034353 - Ramp1	6
14	73835242	ENSMUSG00000036461 - Elf1	6
15	97323751	ENSMUSG00000044250 - NP_758497.1	6
3	1,52E+08	ENSMUSG00000068496 -	6
8	83971639	ENSMUSG00000001911 - NP_035036.1	6
2	6459142	ENSMUSG00000002107 - Cugbp2	6
10	62460764	ENSMUSG000000X077 - Prg1	6
11	1,01E+08	ENSMUSG000000X919 - Stat5b	6
19	4074793	ENSMUSG00000024858 - Adrbk1	6
2	1,56E+08	ENSMUSG00000027636 - Sla2	6
4	1E+08	ENSMUSG00000028530 - Jak1	6
5	1,23E+08	ENSMUSG00000029408 - Abcb9	6
5	1,37E+08	ENSMUSG00000029510 -	6
9	70939914	ENSMUSG00000032X7 - Lipc	6
9	57740572	ENSMUSG00000032312 - Csk	6
18	80729558	ENSMUSG00000033016 - Nfatc1	6
11	74901602	ENSMUSG00000038268 - Ovca2	6
3	1,52E+08	ENSMUSG00000039037 - St6galnac5	6
1	1,78E+08	ENSMUSG00000039630 -	6
12	1,03E+08	ENSMUSG00000048251 - Bcl11b	6
12	1,09E+08	ENSMUSG00000054328 - IGHA_MOUSE	6
1	1,06E+08	ENSMUSG00000057329 - Bcl2	6
14	60680683	ENSMUSG00000059456 - Ptk2b	6
9	61446256	ENSMUSG00000066549 -	6
X	6X0186	ENSMUSG00000068244 -	6
11	1,01E+08	ENSMUSG00000004043 - Stat5a	5
7	61836804	ENSMUSG00000005533 - Igf1r	5
1	1,72E+08	ENSMUSG00000015314 - Slamf6	5
11	1,07E+08	ENSMUSG000000X721 - Helz	5
14	1,13E+08	ENSMUSG00000022132 - Cldn10	5
1	87436768	ENSMUSG00000026288 - Inpp5d	5
2	4218157	ENSMUSG00000026657 - Frmd4a	5
6	8234651	ENSMUSG00000029638 - Glcci1	5
6	1,26E+08	ENSMUSG00000030337 - Q9CXX2_MOUSE	5
7	73986624	ENSMUSG00000030539 - Sema4b	5
7	1,2E+08	ENSMUSG00000030745 - Il21r	5
9	32557874	ENSMUSG0000003X35 - Q8BKG9_MOUSE	5
9	1,21E+08	ENSMUSG00000032536 - 2310001H13Rik	5
11	87392609	ENSMUSG00000034177 - 4732452J19Rik	5
2	1,58E+08	ENSMUSG00000037754 - Ppp1r16b	5
11	1,21E+08	ENSMUSG00000039230 - Tbcd	5
X	1,53E+08	ENSMUSG00000040990 - Sh3kbp1	5
10	42967078	ENSMUSG00000044770 - Scml4	5
9	1,14E+08	ENSMUSG00000045594 - Q8C659_MOUSE	5
11	44370952	ENSMUSG00000057098 - Ebf1	5

19	8185832	ENSMUSG00000059508 - Ahnak	5
15	73753317	ENSMUSG00000059895 - Ptp4a3	5
5	1,16E+08	ENSMUSG00000061288 - A430105I05Rik	5
15	95858141	ENSMUSG00000064210 - Tmem16f	5
5	1,4E+08	ENSMUSG00000000149 - Gna12	5
13	30293541	ENSMUSG00000021357 - Sec5l1	5
19	56618906	ENSMUSG00000025085 - Ablim1	5
1	1,38E+08	ENSMUSG00000026395 - NM_011210.1	5
5	1,22E+08	ENSMUSG00000029475 - Fbxl10	5
6	91455944	ENSMUSG00000030091 - Nup210	5
X	6169063	ENSMUSG00000031161 - Hdac6	5
11	1,18E+08	ENSMUSG00000033909 - Usp36	5
2	62187140	ENSMUSG00000035000 - Dpp4	5
7	92978X4	ENSMUSG00000035354 - Uvrag	5
10	79924769	ENSMUSG00000035835 -	5
2	1,66E+08	ENSMUSG00000039621 - BC067047	5
19	31448X6	ENSMUSG00000040451 - Tmem23	5
13	97856949	ENSMUSG00000041417 - Pik3r1	5
10	95150587	ENSMUSG00000045867 - Cradd	5
9	44500639	ENSMUSG00000047880 - Blr1	5
11	1,08E+08	ENSMUSG00000050965 - Prkca	5
18	55107776	ENSMUSG00000052713 - Zfp608	5
11	1,16E+08	ENSMUSG00000057286 - St6galnac2	5
6	1,26E+08	ENSMUSG00000057311 - Q3TUZ9_MOUSE	5
3	51319671	ENSMUSG00000061143 - Maml3	5
9	61061680	ENSMUSG00000066550 -	5
11	77240781	ENSMUSG00000000686 - 1300007F04Rik	4
17	51101487	ENSMUSG00000000708 - Pcaf	4
3	87886292	ENSMUSG00000001419 - Mef2d	4
2	71983510	ENSMUSG00000004085 - NP_835185.1	4
7	24172878	ENSMUSG00000015149 - Sirt2	4
11	79316936	ENSMUSG00000017639 - Rab11fip4	4
2	1,64E+08	ENSMUSG00000018X9 - Stk4	4
10	28105053	ENSMUSG00000019889 - Ptprk	4
11	1,1E+08	ENSMUSG000000X623 - Map2k6	4
11	67692109	ENSMUSG000000X903 - Stx8	4
12	70432856	ENSMUSG00000021108 - Prkch	4
14	29349699	ENSMUSG00000021904 -	4
14	5524X15	ENSMUSG00000021990 -	4
15	82198134	ENSMUSG00000022463 - Srebf2	4
18	68164044	ENSMUSG00000024544 - D18Ertd653e	4
7	52936661	ENSMUSG00000025324 - Atp10a	4
9	1,09E+08	ENSMUSG00000025647 - Scotin	4
1	1,89E+08	ENSMUSG00000026604 - Ptpn14	4
2	3630730	ENSMUSG00000026655 - 3110001A13Rik	4
2	1,3E+08	ENSMUSG00000027303 - Ptpra	4
4	1,32E+08	ENSMUSG00000028868 - Wasf2	4
5	1,48E+08	ENSMUSG00000029659 - 6330406I15Rik	4
X	18856777	ENSMUSG00000031060 - Rbm10	4
8	94261282	ENSMUSG00000031785 - Gpr56	4
8	1,17E+08	ENSMUSG00000034330 - Plcg2	4
13	52756906	ENSMUSG00000034987 - Hrh2	4
5	3350318	ENSMUSG00000040274 - Cdk6	4
14	48231801	ENSMUSG00000041018 - TCA_MOUSE	4
13	19593015	ENSMUSG00000041112 - Elmo1	4
11	1,15E+08	ENSMUSG00000045980 - C630005D06Rik	4
11	1,19E+08	ENSMUSG00000046697 -	4
15	86265344	ENSMUSG00000051864 - Tbc1d22a	4
15	66694808	ENSMUSG00000053469 - Tgn	4
7	33350807	ENSMUSG00000054676 - 1600014C10Rik	4
15	1,03E+08	ENSMUSG00000060992 -	4
4	1,35E+08	ENSMUSG00000062585 - Cnr2	4
5	1,22E+08	ENSMUSG00000062946 - 4932422M17Rik	4
17	44657989	ENSMUSG00000064043 - Q3TD72_MOUSE	4

5	1,21E+08	ENSMUSG00000064267 - 0610039P13Rik	4
10	69589564	ENSMUSG00000069601 - Ank3	4
11	59952358	ENSMUSG00000000538 - Tom1l2	4
16	88944544	ENSMUSG00000002489 - Tiam1	4
10	80817002	ENSMUSG00000003348 - Mobkl2a	4
6	1,25E+08	ENSMUSG00000004266 - Ptpn6	4
11	1E+08	ENSMUSG00000006931 - 1110036O03Rik	4
2	27044166	ENSMUSG00000009216 - C630035N08Rik	4
11	86311858	ENSMUSG00000018171 - Tmem49	4
10	94765145	ENSMUSG000000X026 - Plxnc1	4
13	50254931	ENSMUSG00000021451 - Sema4d	4
13	51398633	ENSMUSG00000021460 - Auh	4
13	58294595	ENSMUSG00000021556 - Golph2	4
15	34875550	ENSMUSG00000022329 - Stk3	4
15	66805025	ENSMUSG00000022372 - Sla	4
15	77812546	ENSMUSG00000022443 - Myh9	4
18	4336072	ENSMUSG00000024235 - Map3k8	4
17	23616312	ENSMUSG00000025727 -	4
1	16411698	ENSMUSG000000259X - Stau2	4
1	1,6E+08	ENSMUSG00000026721 - 8430421H08Rik	4
2	44918954	ENSMUSG00000026872 - Zfhx1b	4
2	1,32E+08	ENSMUSG00000027339 - Rassf2	4
2	1,26E+08	ENSMUSG00000027366 - X10106G01Rik	4
3	1,34E+08	ENSMUSG00000028163 - Nfkb1	4
4	1,37E+08	ENSMUSG00000028766 - Akp2	4
6	35039352	ENSMUSG00000029847 - Gm468	4
6	99394528	ENSMUSG00000030067 - Foxp1	4
7	1,2E+08	ENSMUSG00000030750 - Nsmce1	4
9	1,12E+08	ENSMUSG00000032503 - Arpp21	4
11	80X7787	ENSMUSG00000035441 - Myo1d	4
17	23782X8	ENSMUSG00000037098 - Rab11fip3	4
8	1,22E+08	ENSMUSG00000037184 -	4
9	63541882	ENSMUSG00000037801 - 4921504K03Rik	4
9	1,22E+08	ENSMUSG00000037949 - Tmem16k	4
4	59259164	ENSMUSG00000038578 - XP_485387.1	4
4	1,48E+08	ENSMUSG00000039936 - Pik3cd	4
11	72177297	ENSMUSG00000040447 - BC011467	4
11	1,06E+08	ENSMUSG00000040699 - 0610025L06Rik	4
1	1,66E+08	ENSMUSG00000040723 - Rcsd1	4
11	5174512	ENSMUSG00000041961 - Q5SSZ7_MOUSE	4
2	70663260	ENSMUSG00000041975 - BC004636	4
17	12883293	ENSMUSG00000046991 -	4
6	1,21E+08	ENSMUSG00000051586 - BC030863	4
1	1,36E+08	ENSMUSG00000052759 -	4
19	46818214	ENSMUSG00000053617 - Sh3md1	4
12	98013455	ENSMUSG00000057963 - Itpk1	4
9	14678688	ENSMUSG00000059658 - XP_486187.1	4
4	1,44E+08	ENSMUSG00000066025 - Vps13d	4
12	1,07E+08	ENSMUSG00000066322 -	4
9	14715316	ENSMUSG00000066933 -	4
2	31906644	ENSMUSG00000001855 - Nup214	3
17	26028726	ENSMUSG00000002250 - Ppard	3
5	1,21E+08	ENSMUSG00000004455 - Ppp1cc	3
1	74539344	ENSMUSG00000006304 - Arpc2	3
2	1,67E+08	ENSMUSG00000006462 - A530013C23Rik	3
19	7953651	ENSMUSG00000010097 - Nxf1	3
1	1,72E+08	ENSMUSG00000015316 - Slamf1	3
13	23807282	ENSMUSG00000016756 - Cmah	3
10	93613732	ENSMUSG000000X019 - Ntn4	3
12	3001830	ENSMUSG000000X661 - Dnmt3a	3
11	1,15E+08	ENSMUSG000000X732 - Rab37	3
5	1,38E+08	ENSMUSG00000021X6 - D830046C22Rik	3
15	85908351	ENSMUSG00000022385 - Gtse1	3
17	76016902	ENSMUSG00000024074 - Crim1	3

18	61445826	ENSMUSG00000024575 - Pde6a	3
19	55323264	ENSMUSG00000024985 - Tcf7l2	3
19	45944266	ENSMUSG00000025231 - Sufu	3
1	1,88E+08	ENSMUSG00000026609 - Q9D1Z8_MOUSE	3
2	38443863	ENSMUSG00000026749 - Nek6	3
2	11088392	ENSMUSG00000026778 - Prkcq	3
2	27188X8	ENSMUSG00000026917 - Wdr5	3
2	68716958	ENSMUSG00000027035 - Lass6	3
2	1,27E+08	ENSMUSG00000027380 - Acoxl	3
3	1,36E+08	ENSMUSG00000028161 - Ppp3ca	3
4	1,19E+08	ENSMUSG00000028634 - Hivep3	3
4	1,32E+08	ENSMUSG00000028874 - Fgr	3
5	1,11E+08	ENSMUSG00000029344 - Tpst2	3
5	1,24E+08	ENSMUSG00000029402 - 6330548G22Rik	3
6	83300099	ENSMUSG00000030041 - D6Mm5e	3
7	95427607	ENSMUSG00000030688 - Stard10	3
X	1,62E+08	ENSMUSG00000031355 - Arhgap6	3
8	24243730	ENSMUSG00000031565 - Q8CBY7_MOUSE	3
8	1,2E+08	ENSMUSG00000031822 - Gse1	3
8	1,06E+08	ENSMUSG00000031924 - 1810044O22Rik	3
8	1,24E+08	ENSMUSG00000031977 - Galnt2	3
9	69585298	ENSMUSG00000032231 - Anxa2	3
9	65336987	ENSMUSG00000032392 - Parp16	3
15	78401171	ENSMUSG00000033287 -	3
10	84517098	ENSMUSG00000034453 - Polr3b	3
12	67971859	ENSMUSG00000034601 - 2700049A03Rik	3
14	68986580	ENSMUSG00000034997 - Htr2a	3
2	1,63E+08	ENSMUSG00000035268 - Pkig	3
19	6770656	ENSMUSG00000036278 - D930010J01Rik	3
17	85139691	ENSMUSG00000036918 - Ttc7	3
11	75257362	ENSMUSG00000038178 - Slc43a2	3
4	57581418	ENSMUSG00000038729 -	3
9	1,19E+08	ENSMUSG00000039115 - Itga9	3
4	62472550	ENSMUSG00000039165 - 4933437N03Rik	3
14	19417297	ENSMUSG00000039197 - Adk	3
17	33636140	ENSMUSG000000392X - NP_787948.1	3
14	19035656	ENSMUSG00000039367 - Sec24c	3
5	1,4E+08	ENSMUSG00000039683 - Sdk1	3
2	29552166	ENSMUSG00000039844 - Rapgef1	3
11	1,07E+08	ENSMUSG00000040528 - Q3TC23_MOUSE	3
19	4003183	ENSMUSG00000040663 - Clcf1	3
3	1,28E+08	ENSMUSG000000412X - Elovl6	3
17	40771665	ENSMUSG00000041293 - Gpr110	3
1	1,36E+08	ENSMUSG00000041642 - Kif21b	3
13	1,1E+08	ENSMUSG00000042348 -	3
15	80625214	ENSMUSG00000042351 - Grap2	3
1	1,53E+08	ENSMUSG00000045372 -	3
5	1,05E+08	ENSMUSG00000046079 - Lrrc5	3
18	65931495	ENSMUSG00000046610 - 5330437I02Rik	3
2	43681004	ENSMUSG00000049744 - Arhgap15	3
3	1,16E+08	ENSMUSG00000051345 -	3
3	67564532	ENSMUSG00000051777 - NP_808253.1	3
13	53979046	ENSMUSG0000005X87 - Rgs14	3
4	82511780	ENSMUSG00000052407 - 4930473A06Rik	3
15	80100799	ENSMUSG00000053457 -	3
3	1,25E+08	ENSMUSG00000053819 - Camk2d	3
8	24327362	ENSMUSG00000054823 - Whsc1l1	3
11	1,03E+08	ENSMUSG00000055805 - Fmnl1	3
17	81539632	ENSMUSG00000055817 - Mta3	3
3	58656487	ENSMUSG00000056476 - Med12l	3
14	73X3233	ENSMUSG00000058997 - 1300010F03Rik	3
15	59384785	ENSMUSG00000059586 - 1110014D18Rik	3
7	X767077	ENSMUSG00000061139 -	3
7	1974X40	ENSMUSG00000061511 -	3

1	85961098	ENSMUSG00000062590 - 4930438O05Rik	3
10	43930653	ENSMUSG00000064118 - XP_618752.1	3
17	33024712	ENSMUSG00000064174 - H2-Q1	3
7	1,38E+08	ENSMUSG00000066096 -	3
12	82224235	ENSMUSG00000066405 -	3
2	1,32E+08	ENSMUSG00000068247 -	3
14	46082381	ENSMUSG00000068415 -	3
3	67553575	ENSMUSG00000068984 -	3
13	30139725	ENSMUSG00000069255 - Dusp22	3
18	151788X	ENSMUSG00000069447 -	3
8	69695402	ENSMUSG00000070002 - Ell	3
10	3464195	ENSMUSG00000000766 - Oprm1	3
7	77499495	ENSMUSG00000001741 - Il16	3
7	1,05E+08	ENSMUSG00000005611 - Mrvi1	3
16	4216162	ENSMUSG00000005718 - Tcfap4	3
10	932X844	ENSMUSG00000008398 - Elk3	3
2	27194271	ENSMUSG00000009621 - Vav2	3
12	1,07E+08	ENSMUSG00000010529 - NP_0010284X.1	3
10	60261433	ENSMUSG00000012819 - Cdh23	3
15	85947573	ENSMUSG00000016028 - Celsr1	3
9	32345966	ENSMUSG00000016087 - Fli1	3
2	1,63E+08	ENSMUSG00000017707 - Tde1	3
6	67544088	ENSMUSG00000018341 - Il12rb2	3
11	1,01E+08	ENSMUSG00000019173 - Rab5c	3
10	1,16E+08	ENSMUSG000000X166 - Cnot2	3
11	29994187	ENSMUSG000000X315 - Spnb2	3
11	80605840	ENSMUSG000000X704 - Accn1	3
11	1,16E+08	ENSMUSG000000X780 - Srp68	3
13	40868457	ENSMUSG00000021365 -	3
13	96973049	ENSMUSG00000021629 - Slc30a5	3
14	19096188	ENSMUSG000000218X - Q3U3H3_MOUSE	3
14	29491719	ENSMUSG00000021892 - Sh3bp5	3
14	64533871	ENSMUSG0000002X92 - Ppp3cc	3
15	97868310	ENSMUSG00000022475 - Q6KAT4_MOUSE	3
16	44921938	ENSMUSG00000022657 - Cd96	3
16	8364074	ENSMUSG00000022710 - Usp7	3
16	57457540	ENSMUSG00000022747 - St3gal6	3
16	93894799	ENSMUSG00000022898 - Dscr3	3
17	49273710	ENSMUSG00000023927 - Satb1	3
19	33824281	ENSMUSG00000024781 - Lip1	3
19	3942782	ENSMUSG00000024830 - Rps6kb2	3
1	37086786	ENSMUSG00000026116 - D1Bwg0491e	3
1	33722765	ENSMUSG00000026134 - Prim2	3
1	1,34E+08	ENSMUSG00000026458 - Ppfia4	3
1	1,52E+08	ENSMUSG00000026482 - Rgl1	3
1	1,67E+08	ENSMUSG00000026558 - Uck2	3
2	91330068	ENSMUSG00000027249 - F2	3
3	1,01E+08	ENSMUSG00000027863 - Cd2	3
4	57005189	ENSMUSG00000028434 - Epb4.1l4b	3
4	86758846	ENSMUSG00000028496 - Mllt3	3
4	1,16E+08	ENSMUSG00000028677 - 4931406IXRik	3
4	1,29E+08	ENSMUSG00000028776 - Tinagl	3
5	34504696	ENSMUSG00000029093 - Sorcs2	3
5	34978351	ENSMUSG00000029192 - Tbc1d14	3
5	71537282	ENSMUSG00000029217 - Tec	3
5	1,07E+08	ENSMUSG00000029270 - 2900024C23Rik	3
5	99572373	ENSMUSG00000029322 - Plac8	3
5	1,39E+08	ENSMUSG00000029554 - Mad1l1	3
5	1,37E+08	ENSMUSG00000029730 - XP_484549.1	3
6	1,44E+08	ENSMUSG00000030283 - St8sia1	3
7	74390860	ENSMUSG00000030527 - 2610312FXRik	3
7	1,22E+08	ENSMUSG00000030844 - Rgs10	3
7	98521386	ENSMUSG00000030921 - Trim30	3
X	51986X2	ENSMUSG00000031133 - Arhgef6	3

X	1,28E+08	ENSMUSG00000031264 - Btk	3
8	12945586	ENSMUSG00000031453 - Rasa3	3
8	95145470	ENSMUSG00000031672 - Got2	3
9	40966048	ENSMUSG0000003XX - 2810457I06Rik	3
9	63767270	ENSMUSG00000032402 - Smad3	3
6	87568335	ENSMUSG000000334X - Antxr1	3
12	95744449	ENSMUSG00000033530 -	3
12	94622266	ENSMUSG00000033713 - Ches1	3
8	1,19E+08	ENSMUSG00000034189 - Hsdl1	3
9	51362426	ENSMUSG00000036027 - 1810046K07Rik	3
17	21982358	ENSMUSG00000036473 - C530046L02Rik	3
13	48400248	ENSMUSG00000038025 - Phf2	3
8	10296139	ENSMUSG00000038917 - 3930402G23Rik	3
4	62451938	ENSMUSG00000039158 - AI597013	3
17	47524721	ENSMUSG00000039316 - 2310015N21Rik	3
18	64763659	ENSMUSG00000039529 - Atp8b1	3
10	30688332	ENSMUSG00000039697 - Ncoa7	3
3	1,2E+08	ENSMUSG00000039865 - Slc44a3	3
10	1,28E+08	ENSMUSG00000039914 -	3
7	39972419	ENSMUSG00000040231 -	3
16	93527067	ENSMUSG000000408X - Q3TG10_MOUSE	3
6	1,45E+08	ENSMUSG00000041540 -	3
11	1,13E+08	ENSMUSG00000041654 - Slc39a11	3
8	86945303	ENSMUSG00000045333 - Zfp423	3
5	1,37E+08	ENSMUSG00000045348 - 6430598A04Rik	3
7	1,37E+08	ENSMUSG00000045777 - 6330512M04Rik	3
16	29764502	ENSMUSG00000047434 - AI480653	3
15	72616616	ENSMUSG00000047921 - 1810044A24Rik	3
6	72717198	ENSMUSG00000050732 - Vamp8	3
16	563X608	ENSMUSG00000051628 - 2610528E23Rik	3
19	32466225	ENSMUSG0000005X09 - 6530404N21Rik	3
9	1,2E+08	ENSMUSG00000052336 - Cx3cr1	3
18	75666391	ENSMUSG00000052928 - Gm672	3
4	56847335	ENSMUSG00000055296 - NP_780727.2	3
12	73X5034	ENSMUSG00000056459 - Zbtb25	3
9	1,22E+08	ENSMUSG00000060595 -	3
14	1,16E+08	ENSMUSG00000063410 - Stk24	3
6	52913123	ENSMUSG00000063568 - AI591476	3
5	1,39E+08	ENSMUSG00000066661 -	3
7	23753815	ENSMUSG00000066674 -	3
6	87869211	ENSMUSG00000068266 -	3
16	15619694	ENSMUSG00000068589 -	3
11	58130822	ENSMUSG00000069871 -	3

Supplemental Methods for CGH comparison

We obtained 10K SNP array CGH data for 713 human cancer cell lines from the Wellcome Trust Sanger Institute (ftp://ftp.sanger.ac.uk/pub/CGP/10kData). Regions of copy number change were identified using DNAcopy version 1.4.0 (Olshen et al., 2004) and MergeLevels (Willenbrock and Fridlyand, 2005). Both packages are available for R from BioConductor (http://www.bioconductor.org). DNAcopy was used to detect change-points in copy number data by circular binary segmentation. Log_2-ratios were smoothed to remove outliers, chromosomes were segmented using default parameters, and change-points less than 3 SDs apart were removed. MergeLevels was used with default parameters to merge the segmentation results into a defined set of copy number levels. For each cell line, the predicted log_2-ratio closest to 0 was defined as the level of no copy number change and, to enable comparison across cell lines, this log_2-ratio was set to 0 and all other log_2-ratios were normalized accordingly. Copy numbers are the ratio of tumour/normal intensities i.e. a copy number of 1 represents 2 DNA copies in a normal, diploid cell. Our analysis was limited to amplicons and deletions smaller than 70Mb. To test for overlap between CIS loci and amplicons we used the human orthologs of CIS genes and compared them to the recurrently amplified regions of the autosomal chromosomes in the CGH data (i.e. copy number 1.5 or above in 4 or more cell lines). The genomic coordinates of the human orthologs were extracted from Ensembl v37 (Hubbard et al., 2007) using BioMart (http://www.biomart.org). Orthologs within or overlapping the boundaries of gains and losses in the human cancer cell lines were identified. The boundaries were defined as 500 kb upstream of the start of the corresponding segment, or 1 bp downstream of the end of the previous segment, whichever is closer, and 500 kb downstream of the end of the corresponding segment, or 1 bp upstream of the start of the next segment, whichever is closer. To determine the significance of the overlap, all mouse genes with human orthologs in Ensembl were placed in a 2x2 contingency table based on whether the gene is found within the set of CIS genes and whether the ortholog is amplified (to copy number 1.5 or above in 4 or more cell lines). The p-value was calculated using a one-tailed Fisher's exact test.

Supplemental References

Hubbard,T.J., Aken,B.L., Beal,K., Ballester,B., Caccamo,M., Chen,Y., Clarke,L., Coates,G., Cunningham,F., Cutts,T., et al. (2007). Ensembl 2007. Nucleic Acids Res *35*, D610-D617.

Olshen,A.B., Venkatraman,E.S., Lucito,R., and Wigler,M. (2004). Circular binary segmentation for the analysis of array-based DNA copy number data. Biostatistics. *5*, 557-572.

Willenbrock,H. and Fridlyand,J. (2005). A comparison study: applying segmentation to array CGH data for downstream analyses. Bioinformatics *21*, 4084-4091.
